# Supplementary material for: Catalytic inverse vulcanization
Source: Nat Commun. 2019 Feb 7;10:647. doi: 10.1038/s41467-019-08430-8 (PMC6367372; doi:10.1038/s41467-019-08430-8)
Supplement: Supplementary file 1 — Supplementary Information [file 41467_2019_8430_MOESM1_ESM.pdf]

Supplementary Information

Catalytic Inverse Vulcanisation

Xiaofeng Wu *et al.*

Supplementary methods

Materials Sulfur (S_8, sublimed powder, reagent grade, ≥99.5 %, Brenntag UK & Ireland. Purchased in 25 Kg bags), ethylene glycol dimethylacrylate (EGDMA, 98%, Alfa Aesar), glyoxal bis(diallyl acetal) (GBDA, Aldrich), *trans,trans,cis*-1,5,9-cyclododecatriene (CDDT, 98%, Alfa Aesar), 1,3,5,7-tetravinyltetramethylcyclotetrasiloxane (TVTMCTSi, 97%, Alfa Aesar), 1,2,4-trivinylcycohexane (TVCH, 98%, Fluorochem), dicyclopentadiene (DCPD, >95%, TCI), 1,3-diisopropenylbenzene (DIB, 97%, Aldrich), divinylbenzene (DVB, 80%, Merck), (*R*)-(+)-limonene (97%, Aldrich), squalene (≥98 %, Alfa Aesar), linseed oil (Aldrich), sunflower oil (Tesco), sodium diethyldithiocaebamate trihydrate (Alfa Aesar), copper diethyldithiocaebamate (TCI), nickel diethyldithiocaebamate (TCI), ZnO (Aldrich), zinc (Aldrich), $ZnCl_2$ (Aldrich), $FeCl_2$ (Aldrich), CuO (Aldrich), $CuCl_2$ (Aldrich), zinc stearate (Aldrich), 2-Cyano-2-propyl benzodithioate (>97%, Aldrich), thiram (Aldrich), chloroform (Aldrich), and chloroform-d ($CDCl_3$,Cambridge Isotope Laboratories Inc.) were commercially available and used as received without any further purification. Iron diethyldithiocaebamate and cobalt diethyldithiocaebamate were both synthesized from sodium diethyldithiocaebamate following a method reported in the literature.[1]

Instrumentation used for characterization:

Gel permeation chromatography (GPC): The molecular weight of the soluble fraction of the polymers was determined by gel permeation chromatography (GPC) using a Viscotek system comprising a GPCmax (degasser, eluent and sample delivery system), and a TDA302 detector array, using THF as eluent.

Powder X-ray Diffraction (PXRD): Data was measured using a PANalytical X'Pert PRO diffractometer with Cu-$K_{\alpha1+2}$ radiation, operating in transmission geometry.

Differential Scanning Calorimetry (DSC) were performed on a TA Instruments Q200 DSC, under nitrogen flow, and with heating and cooling rates of 5 °C/min.

Thermogravimetric analysis (TGA) samples were heated under nitrogen to 800 °C at a heating rate of 20 °C min^{-1} using a TA Instruments Q500.

Fourier-transform infrared spectroscopy (FT-IR) was performed using a Thermo NICOLET IR200, between 400 cm^{-1} to 4000 cm^{-1}. Samples were loaded either neat, using an attenuated total reflectance accessory, or in transmission after pressing into a KBr pellet.

Solution NMR was recorded in deuterated chloroform using a Bruker Advance DRX (400 MHz) spectrometer.

^{13}C magic-angle spinning (MAS) NMR spectra were performed on a Bruker Avance III operating at a ^{1}H Larmor frequency of 700 MHz, using a Bruker 4mm HX probe. Chemical shifts were referenced using the CH_3 resonance of solid alanine at 20.5 ppm (^{13}C). A chemical shielding reference of 189.7 ppm was used, determined from a separate calculation on an optimized tetramethylsilane molecule.

Experimental procedures:

A note on the reproducibility and sensitivity of inverse vulcanization reactions: We have noted that these reactions are particularly susceptible to changes in apparatus and conditions. This results from the nature of the reactions, being driven by radical initiation, and exothermic polymerization. Care must be taken not to let the temperature of the heating medium (we use metal heating blocks on hot plate stirrers) to 'overshoot' in temperature, as this will affect the reaction time. The sulfur polymerization mixture is also quite a poor thermal conductor, and if care is not taken, the exothermic reaction causes the internal temperature to increase above the intended temperature. The reaction will therefore proceed differently depending on the heat transfer away from the reaction. As a result, using different sizes and shapes of heating blocks, different glassware volumes, stirrer speeds, stirrer geometries etc. will all affect this process. For this reason, we took great care to keep all of these variables constant in these reactions.

General procedure for the catalysts discovery and screening: preparation of poly (sulfur-*random*-(ethylene glycol dimethacrylate)) (Poly(S-*r*-EGDMA))

To a 40 mL glass reaction vial equipped with a magnetic stir bar was added 5 g (19.5 mmol) of elemental sulfur, catalysts (masses detailed below) and heated until molten by placing the vial in a metal heating block set to 135 °C. The melting point of sulfur is ~120 °C. The reactions were stirred at 200 RPM using cross shaped magnetic stirrer bars. When the sulfur was molten, 5 g (25.2 mmol) of Ethylene glycol dimethacrylate (EGDMA) cross-linker was added. The stirring rate was then increased to 900 RPM,

and the reaction continued for up to 10 hours. Samples that were observed to react to form a homogeneous molten state (does not separate if removed on a spatula and cooled to room temperature), were then removed from stirring and cured in an oven at 140 °C for 10 hours further. Samples that showed no sign of reaction, and that were still two phases after 10 hours were aborted.

Preparation of Poly(S-*r*-EGDMA) with 1 w% of ZnO as catalyst: The copolymerization was carried out by the following the general method mentioned above with ZnO (100 mg, 1 w% loading, 1.22 mmol) to afford two layers of mixture with yellow solid at bottom and a clear liquid on the top (yield: 9.7 g). Elemental Analysis for $(C_{10}H_{14}O_4+S_8)_n$ (50 w% S), Calcul. (%): C, 32.95; H, 3.56; S, 50; Found: C, 29.52; H, 3.30; S, 49.58. PXRD and DSC confirmed the presence of unreacted sulfur.

Preparation of Poly(S-*r*-EGDMA) with 1 w% of Zinc as catalyst: The copolymerization was carried out by the following the general method mentioned above with Zinc (100 mg, 1w% loading, 1.53 mmol) to afford two layers of mixture with yellow solid at bottom and a clear liquid on the top (yield: 9.3 g). PXRD and DSC confirmed the presence of unreacted sulfur.

Preparation of Poly(S-*r*-EGDMA) with 1 w% of Zinc Chloride as catalyst: The copolymerization was carried out by the following the general method mentioned above with $ZnCl_2$ (100 mg, 1w% loading, 0.736 mmol) to afford two layers with gray-brown solid at the bottom and a clear liquid above (yield: 9.5 g). PXRD and DSC confirmed the presence of unreacted sulfur.

Preparation of Poly(S-*r*-EGDMA) with 1 w% of iron Chloride as catalyst: The copolymerization was carried out by the following the general method mentioned above with $FeCl_2$ (100 mg, 1w% loading, 0.787 mmol) to afford two layers with brown-red solid at the bottom and clear liquid on above (yield: 9.3 g). PXRD and DSC confirmed the presence of unreacted sulfur.

Preparation of Poly(S-*r*-EGDMA) with 1 w% of Copper oxide as catalyst: The copolymerization was carried out by the following the general method mentioned above with CuO (100 mg, 1w% loading, 1.26 mmol) to afford two layers of mixture with brown solid at bottom and liquid on the top (yield: 9.1 g). PXRD and DSC confirmed the presence of unreacted sulfur.

Preparation of Poly(S-*r*-EGDMA) with 1 w% of Copper Chloride as catalyst: The copolymerization was carried out by the following the general method mentioned above with $CuCl_2$ (100 mg, 1w% loading, 0.743 mmol) to afford two layers with a brown-green solid at the bottom and a clear liquid above (yield: 9.3 g). PXRD and DSC confirmed the presence of unreacted sulfur.

Preparation of Poly(S-*r*-EGDMA) with 1 w% of Zinc Stearate (Zn-STR) as catalyst: The copolymerization was carried out by the following the general method mentioned above with Zn-STR (100 mg, 1w% loading, 0.743 mmol) to afford an orange -red solution that cooled to a solid (yield: 9.3 g). Elemental Analysis for $(C_{10}H_{14}O_4+S_8)_n$ (50 w% S), Calcul. (%): C, 32.95; H, 3.56; S, 50; Found: C, 31.75; H, 3.64; S, 47.33. PXRD and DSC confirmed the presence of unreacted sulfur.

Preparation of Poly(S-*r*-EGDMA) with 1 w% of Zinc diethyldithiocarbamate (ZnD_2) as catalyst: The copolymerization was carried out by the following the general method mentioned above with ZnD_2 (100 mg, 1w% loading, 0.276 mmol) to afford a dark-red homogeneous gel and then black-red solid (yield: 9.9 g). Elemental Analysis for $(C_{10}H_{14}O_4+S_8)_n$ (50 w% S), Calcul. (%): C, 32.95; H, 3.56; S, 50; Found: C, 28.72; H, 3.25; S, 51.48. DSC and PXRD confirm the absence of crystalline S_8. The T_g was 20 °C.

Preparation of Poly(S-*r*-EGDMA) with 1 w% of Iron diethyldithiocarbamate (Fe-D) as catalyst: The copolymerization was carried out by the following the general method mentioned above with Fe-D (100 mg, 1w% loading, 0.284 mmol) to afford a black-green homogeneous gel and then a black solid (yield: 9.8 g). Elemental Analysis for $(C_{10}H_{14}O_4+S_8)_n$ (50 w% S), Calcul. (%): C, 32.95; H, 3.56; S, 50; Found: C, 29.73; H, 3.29; S, 49.99. DSC and PXRD confirm the absence of crystalline S_8. The T_g was 22 °C.

Preparation of Poly(S-*r*-EGDMA) with 1 w% of Cobalt diethyldithiocarbamate (Co-D) as catalyst: The copolymerization was carried out by the following the general method mentioned above with Co-D (100 mg, 1w% loading, 0.281 mmol) to afford a dark-brown homogeneous gel and then a black solid (yield: 9.6 g). Elemental Analysis for $(C_{10}H_{14}O_4+S_8)_n$ (50 w% S), Calcul. (%): C, 32.95; H, 3.56; S, 50; Found: C, 28.89; H, 3.29; S, 51.10. DSC and PXRD confirm the absence of crystalline S_8. The T_g was 18 °C.

Preparation of Poly(S-*r*-EGDMA) with 1 w% of Copper diethyldithiocarbamate (Cu-D) as catalyst: The copolymerization was carried out by the following the general method mentioned above with Cu-D (100 mg, 1w% loading, 0.278 mmol) to afford a dark-red homogeneous gel and then a black-red solid (yield: 9.8 g). Elemental Analysis for $(C_{10}H_{14}O_4+S_8)_n$ (50 w% S), Calcul. (%): C, 32.95; H, 3.56; S, 50; Found: C, 30.07; H, 3.39; S, 49.49. A slight melting transition was detected for unreacted S_8 by DSC, but no crystallinity was detected by PXRD. The T_g was 24 °C.

Preparation of Poly(S-*r*-EGDMA) with 1 w% of Nickel diethyldithiocarbamate (Ni-D) as catalyst: The copolymerization was carried out by the following the general method mentioned above with Ni-D (100 mg, 1w%

loading, 0.282 mmol) to afford a dark-brown homogeneous gel and then a black solid (yield: 9.7 g). Elemental Analysis for $(C_{10}H_{14}O_4+S_8)_n$ (50 w% S), Calcul. (%): C, 32.95; H, 3.56; S, 50; Found: C, 29.63; H, 3.38; S, 50.13. DSC and PXRD confirm the absence of crystalline S_8. The T_g was 18 °C.

Synthesis of sulfur polymers with a range of crosslinkers, with and without ZnD_2 catalyst: To a 40 mL glass reaction vial equipped with a cross shaped magnetic stir bar was added 5 g (19.5 mmol) of elemental sulfur, 5 g of crosslinker, and Zn-Diethyldithiocarbamate catalyst (0 mg, 100 mg, or 500 mg) and heated until molten by placing the vial in a metal heating block set to 135 °C. The melting point of sulfur is ~120 °C. A rubber septum was placed over the top of the vial, and pierced with a needle to allow outgassing during heating. After the first 10 minutes, by which time the sample was completely molten, the needle was removed and the stirring increased to 900 RPM. The use of a rubber septum reduces loss of monomers by evaporation. The high stirring rate aids heat transfer to the sides of the reaction to prevent increases in temperature due to the exothermic reaction. The end-point of the reaction was taken as the point at which solidification of the reaction mixture caused the stirrer bar to cease motion. However, all reactions were left in the heating blocks for at least 24 hours before being removed and allowed to cool. The reactions were monitored for the first hour, and then in half hour intervals for the first 12 hours, then checked again after 24 hours (hence why the reaction time is listed as 12-24 hours for some reactions). After cooling samples were recovered by breaking the vials. All of these reactions were performed in triplicate to ensure the timings were consistent, and allow DSC to be performed on three separate reactions. All crosslinkers were prepared according to the above method, except for limonene, which is known to produce low molecular weight byproducts including cymene, where the reaction was performed under vacuum distillation as reported by Chalker *et al.*[2].

Synthesis of moulded objects: Moulded objects (such as the 'robots' in Supplementary figure 2) were produced using a modified version of the synthesis above. The initial phase of the reaction was the same, but when the reaction mixture had formed a 'pre-polymer' state (increase in viscosity, darkened colour, single phase, no separation on cooling) it was transferred from the glass vial into a silicone mould and placed in an over at 140 °C for a further 12 hours to cure.

General procedure for the synthesis of sulfur-rich polymers with combined cross-linkers by using Zn Diethyldithiocarbamate as catalyst: To a 40 mL glass reaction vial equipped with a magnetic stir bar was added 5 g (19.5 mmol) of elemental sulfur, Zn Diethyldithiocarbamate (100 mg, 1 w% loading, 0.276 mmol) and heated until

molten by placing the vial in a metal heating block set to 135 °C. The melting point of sulfur is ~120 °C. The reactions were stirred vigorously using magnetic stirrer bars. When the sulfur was molten, 5 g of combined two cross-linkers (masses detailed below) were added. The reaction time was counted from the addition of cross-linker to the molten sulfur, until the reaction was aborted or a homogeneous sSupplementary table polymer was formed. Formation of a sSupplementary table homogeneous polymer was indicated by gelation and darkening of the reaction mixture, or solid of the reaction mixture. Homogeneous polymer samples were then transferred into an oven to cure. The oven was pre-heated to 140 °C and the sample cured at this temperature for 10 h before being cooled to room temperature and removed.

Preparation of poly (sulfur-*random*-TVTCSi & CDDT) Poly(S-*r*-TVTCSi/CDDT) with 1 w% of Zinc diethyldithiocarbamate as catalyst: The copolymerization was carried out by the following the general method mentioned above in the presence of catalysts with two cross-linkers of TVTCSi (2.5 g, 7.34 mmol) and CDDT (2.5 g, 15.41 mmol) to afford a red homogeneous gel and black solid (yield: 9.9 g). Elemental Analysis for $(C_{24}H_{42}O_4Si_4+S_8)_n$ (50 w% S), Calcul. (%): C, 28.43; H, 4.17; S, 50; Found: C, 31.28; H, 4.17; S, 52.01.

Preparation of poly (sulfur-*random*-TVTCSi & DCPD)) Poly(S-*r*-TVTCSi/DCPD) with 1 w% of Zinc diethyldithiocarbamate as catalyst: The copolymerization was carried out by the following the general method mentioned above in the presence of catalysts with two cross-linkers of TVTCSi (2.5 g, 7.34 mmol) and DCPD (2.5 g, 18.91 mmol) to afford a red homogeneous gel and black solid (yield: 10.0 g). Elemental Analysis for $(C_{22}H_{36}O_4Si_4+S_8)_n$ (50 w% S), Calcul. (%): C, 27.71; H, 3.81; S, 50; Found: C, 32.60; H, 3.88; S, 50.20.

Preparation of poly (sulfur-*random*-TVTCSi & EGDMA)) Poly(S-*r*-TVTCSi/EGDMA) with 1 w% of Zinc diethyldithiocarbamate as catalyst: The copolymerization was carried out by the following the general method mentioned above in the presence of catalysts with two cross-linkers of TVTCSi (2.5 g, 7.34 mmol) and EGDMA (2.5 g, 12.6 mmol) to afford a red homogeneous gel and black solid (yield: 9.7 g). Elemental Analysis for $(C_{22}H_{38}O_8Si_4+S_8)_n$ (50 w% S), Calcul. (%): C, 24.34; H, 3.53; S, 50; Found: C, 24.49; H, 3.36; S, 50.14.

Preparation of poly (sulfur-*random*-TVTCSi & Farnesol)) Poly(S-*r*-TVTCSi/Farnesol) with 1 w% of Zinc diethyldithiocarbamate as catalyst: The copolymerization was carried out by the following the general method mentioned above in the presence of catalysts with two cross-linkers of TVTCSi (2.5 g, 7.34 mmol) and Farnesol (2.5 g, 11.24 mmol) to afford a red homogeneous gel and black-red solid (yield: 9.2 g). Elemental Analysis for $(C_{27}H_{50}O_5Si_4+S_8)_n$ (50 w% S), Calcul. (%): C, 28.59; H, 4.45; S, 50; Found: C, 29.30; H, 4.35; S, 52.36.

Experimental for the H_2S gas determination

General procedure for catalytic inverse vulcanization: In a glove box, Sulfur (5 g, 19.5 mmol), cross-linkers (5 g), and ZnD_2 catalyst (100 mg, 0.276 mmol) were added to a 40 mL reaction vial equipped with a stirrer bar under N_2 atmosphere. The vial was sealed with a rubber septum and the reaction was setup in a fume hood. The vial was then connected with a N_2-degassed tube ended with a needle to a measuring cylinder (100 mL). The measuring cylinder was filled with deionised water and was placed upside down in a 1 L beaker with water. The reaction mixture was heated until molten by placing the vial in a metal heating block set to 135 °C. The reactions were stirred vigorously using magnetic stirrer bars for each crosslinkers until each reaction produced no more gas, typically under an hour.

General procedure for non-catalytic inverse vulcanization: In a glove box, Sulfur (5 g, 19.5 mmol) and cross-linkers (5 g) were added to a 40 mL reaction vial equipped with a stirrer bar under N_2 atmosphere. The vial was sealed with a rubber septum and the reaction was setup in the normal fume hood. The vial was then connected with a N_2-degassed tube ended with a needle to a measuring cylinder (100 mL). The measuring cylinder was filled with deionised water and was placed upside down in a 1 L beaker with water. The reaction mixture was heated until molten by placing the vial in a metal heating block set to normally higher temperatures that the equivalent reactions without catalysts, to induce similar degrees of reaction over the same timescale. Temperatures were therefore chosen according to the relative reactivities of the crosslinkers and were as follows: Limonene 180 °C, DCPD 170 °C, ENB 135 °C, DVB and DIB 160 °C. The reactions were stirred vigorously using magnetic stirrer bars for each crosslinkers until each reaction produced no more gas, typically under an hour. Another experiment was performed in the same manner with limonene, at 1 wt.% ZnD_2 loading, but at 180 °C, the same temperature as the uncatalysed reaction.

Observations: The reaction of sulfur and limonene without catalyst generated 63 mL H_2S gas, while with 1 wt.% catalyst, the same reaction only generated 10 mL of the H_2S gas. The former reaction was carried out at 180 °C for 15 min and the latter at 135 °C for 50 min. Similar observations were reached for DCPD, for which the reaction was carried out at 170 °C in the absence of catalysts for 27 min, the reaction produced 26.5 mL of gas. The same reaction with 1 wt.% catalyst was carried out at 135 °C for 45 min, the amount of gas generated was only 3.5 mL. The generation of H_2S gas is dependent on the presence of α-proton of allyl groups and related to the reaction temperature. Therefore, with those cross-linkers without α-proton of allyl groups (e.g. DVB, DIB), lower amounts of gas were released, and only at the beginning of the

reaction, there was not any gas generated after the first 3-5 min, especially for the reactions with catalysts. ENB is more reactive than DCPD, and as a result requires lower temperatures, producing less H_2S. In the cases only small amounts of gas were produced (few mL from 10 g reactions), it is possible this recorded volume is at least partly the result of the desorption of gases and moisture dissolved in the reactants, because of heating. In the samples were larger amounts of gas were produced (DCPD and limonene without catalyst), the production of H_2S was confirmed by exposing a H_2S detector to the gas produced.

Heavy Metal Remediation Testing

Synthesis of polymer coated fumed silica: In a round bottom flask, 0.5 g of S-limonene polymers was dissolved in 50 mL of tetrahydrofuran (THF) at room temperature. 5 g of silica gel (Fluorochem, pore size 60 Å, 40-63 µm particle size) was added then added to the flask and the mixture agitated for several minutes. THF was removed by placing the round bottom flask on a rotary evaporator (water bath at 45 °C) and the material was evaporated to dryness, at which point the flask was removed and the coated silica particles were weighed and stored in a glass vial.

Effect of catalyst loading on metal uptake: To determine whether the amount of catalyst used in the sulfur polymer synthesis affected the amount of metal removed from an aqueous solution, polymer coated silica samples were prepared from polymers synthesised with 0, 1 and 5 wt.% catalyst (ZnD_2) loading. To test the effect on metal uptake, 400 ppm solutions of Hg, Au, and Fe were prepared. Each test involved placing 240 mg of coated silica in 12 mL of chosen metal solution, capping the vial and agitating the vials on a tube roller for 60 minutes. After 1 hour the samples were removed, filtered using 0.45 µm nylon syringe filter and an aliquot from each was removed for ICP-OES analysis. Metal uptake was calculated by difference when compared to a control sample which did not contain the coated silica.

Kinetic and capacity studies using mercury solution: Before conducting capacity tests for the sorbent, a kinetics study was preformed to determine the optimal time to leave the samples to fully adsorb the mercury from an aqueous solution. The kinetics study was performed by placing 240 mg of 5% catalysed S-Lim coated silica in to vials with 12 mL of 1000 ppm mercury chloride aqueous solution. Once capped the vials were placed on a tube roller for agitation before being removed at regular intervals. Once a sample was removed, a small aliquot was removed, diluted by a factor 1:100 and analysed by ICP-OES. Mercury uptake was calculated by difference when compared to a control sample which did not contain the 5% S-Lim coated silica. A

capacity study was conducted by sampling different mass of 5% S-Lim coated silica (120, or 240 mg) in different mercury chloride solution strengths (125 – 2000 ppm). A peak capacity for the material was calculated at 65.250 mg of mercury per gram of sorbent used. However the majority of the sorbent mass is the fumed silica support, suggesting that the capacity of the polymer is likely to be x10 greater than the capacity for the whole polymer coated silica. To ensure that the silica itself played no part in the mercury removal process a control of uncoated fumed silica was added to 12 mL of 1000 ppm mercury solution and agitated for 16 hours on a tube roller as well. Negligible mercury capture was noted (<3%).

Affinity for other metals: To test the selectivity and ability of the 5% S-Lim coated silica, a range of different metals were tested. These included gold chloride, methylmercury chloride (to simulate organomercury compounds), cadmium chloride, chromium chloride iron (III) chloride and lead nitrate. All solutions were prepared to 100 ppm concentration. Tests involved using 240 mg of 5% catalyst S-Limonene and 12 mL of the test solution. Samples were then capped and agitated on a tube roller for 16 hours, after which an aliquot of solution was removed, filtered and analysed via ICP-OES. Metal uptake was calculated by difference when compared to a control sample which did not contain the coated silica.

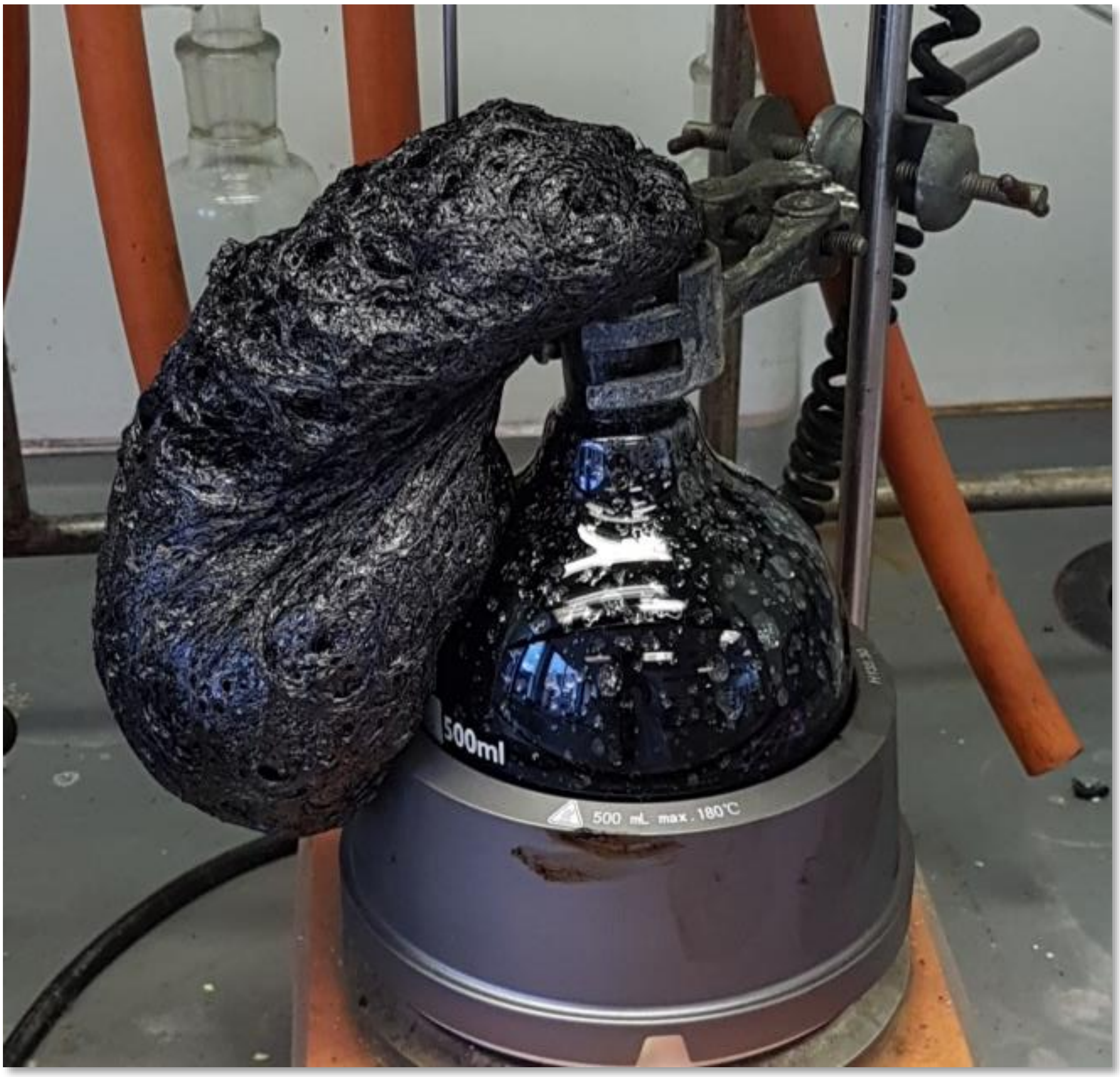

Supplementary figure 1: An example of an inverse vulcanization reaction that has undergone unintended auto-acceleration. In this case, Sulfur-DCPD copolymer. During polymerization, the liquid reaction mixture filled less than 100 mL of the 500 mL round bottom flask as a calm black liquid. Within a few seconds it boiled up violently to overflow the container, venting gas and volatilized monomer, before rapidly setting as a solid.

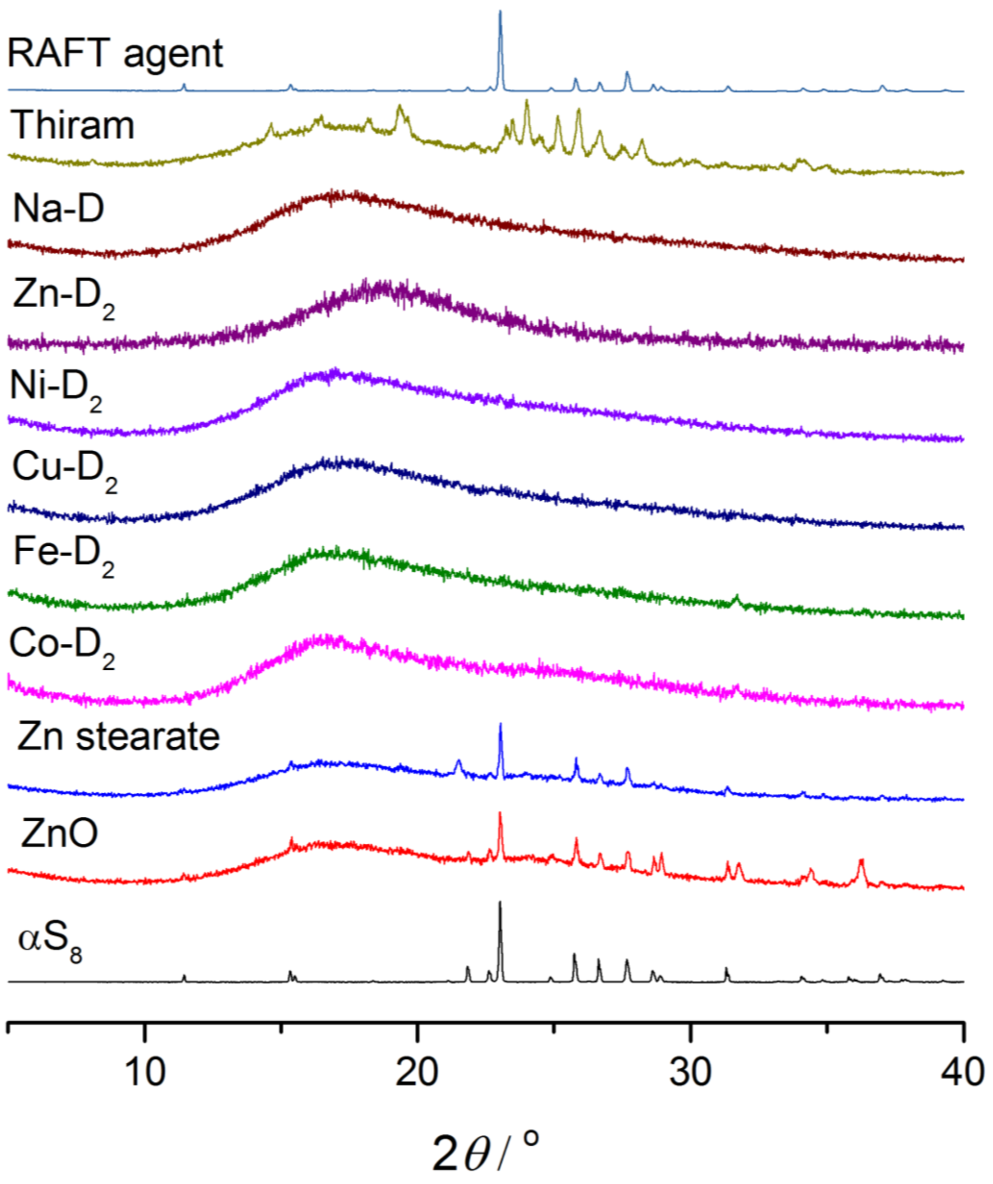

Supplementary figure 2. PXRD diffraction of the products of sulfur reacted with EGDMA (1:1 mass ratio, 135 °C, 10 hours with stirring, followed by a 10 hour cure at 140 °C. Catalyst loading was 1 wt. %. Zinc oxide and Zn stearate catalysts show residual S_8 crystals still present. All diethyldithiocarbamate catalysts (-D) were amorphous, indicating the absence of depolymerized S_8 crystals. Polymerisation of EGDMA occurred with thiram, but poor mixing and incomplete incorporation of the sulfur, with γS_8 crystals evident. The RAFT agent used was 2-Cyano-2-propyl benzodithioate, but showed no inclusion of the sulfur.

Supplementary table 1. Time taken from the start of reaction, until solidification of the reaction mixture causes the stirrer bar to cease. All reactions at 135 °C unless stated, all reactions performed with an equal crosslinker to sulfur mass, and on a 10 g scale, and in triplicate. The reactions were typically monitored for the first hour, and then in half hour intervals for the first 12 hours, then checked again after 24 hours (hence why the reaction time is listed as between 12 and 24 hours for some reactions).

	**Time until solidification, hours**			
**Crosslinker**	**0 wt. % ZnD_2**	**1 wt. % ZnD_2**	**5 wt. % ZnD_2**	**Comments**
DIB	>12, <24	6.5	1	
Limonene	20	8.5	1	
DVB	3.5	1.5	0.85	
DCPD	>12, <24	1.6	0.4	
Squalene	>12, <24	>12 (<24)	1.5	
Sunflower oil	>24	>12 (<24)	<2	S8 detected in all, 0% catalyst reaction still liquid after 24 hr
linseed	>24	4	2.5	S8 detected at 0%, and trace S8 at 5% catalyst, 0% catalyst reaction still liquid after 24 hr
VNB	>12, <24	>4, <6	2	
CDDT	>12, <24	6	3	Both CDDT and TVCH, at O% catalyst loading, did react to form polymeric material, but with significant evaporation of the crosslinker, low yields, and unreacted sulfur sublimed on the sides of the vessel.
TVCH	>12, <24	6	2	
TVTCSi	No reaction	3.5	1.75	Reacted at 160 °C (No reaction at any catalyst loading at 135 °C
EDGMA	No reaction	>12, <24	>12, <24	0 % looks like sulfur
GBDA	>36, <48	>12, <24	2.5	0% catalyst loading reaction is prohibitively slow, taking over 36 hours to form a solid

Supplementary table 2: DSC allows the success of the reaction at stabilising the polymeric to be determined by detecting the melting transition of S_8 crystals. Samples and conditions correspond to those listed in Supplementary table 1. Reaction temperatures are 135 °C unless stated otherwise. * No S_8 crystals were detected in the lower phase of the uncatalysed GBDA reaction, but the extremely long reaction time required for gelation (over 36 hours) led to significant sublimation of crystalline sulfur above this.

Crosslinker	**S_8 melting detected by DSC?**		
	**0 wt. % ZnD_2**	**1 wt. % ZnD_2**	**5 wt. % ZnD_2**
DIB	No	No	No
Limonene	No	No	No
DVB	No	No	No
DCPD	No	No	No
Squalene	No	No	Yes - slight
Sunflower oil	Yes	Yes	Yes
linseed	Yes	No	No
VNB	No	No	No
CDDT	Yes	No	No
TVCH	No	No	No
TVTCSi (135 °C)	Yes	Yes	Yes
TVTCSi (160 C)	Yes	No	No
EDGMA	Yes	No	No
GBDA	No*	No	No

Supplementary table 3: PXRD allows the success of the reaction at stabilising the polymeric to be determined by detecting diffraction from crystalline S_8. Samples and conditions correspond to those listed in Supplementary table 1. Reaction temperatures are 135 °C unless stated otherwise. * No S_8 crystals were detected in the lower phase of the uncatalysed GBDA reaction, but the extremely long reaction time required for gelation (over 36 hours) led to significant sublimation of crystalline sulfur above this.

Crosslinker	**S_8 crystals detected by PXRD?**		
	**0 wt. % ZnD_2**	**1 wt. % ZnD_2**	**5 wt. % ZnD_2**
DIB	No	No	No
Limonene	No	No	No
DVB	No	No	No
DCPD	No	No	No
Squalene	No	No	No
Sunflower oil	Yes αS_8	Yes αS_8	Yes αS_8
linseed	Yes αS_8	No	Yes – slight αS_8

VNB	No	No	No
CDDT	No	No	No
TVCH	No	No	No
TVTCSi (135 °C)	Yes αS_8	Yes αS_8	Yes αS_8
TVTCSi (160 C)	Yes αS_8	No	Yes – very slight trace of γS_8
EDGMA	Yes	No	No
GBDA	No*	No	No

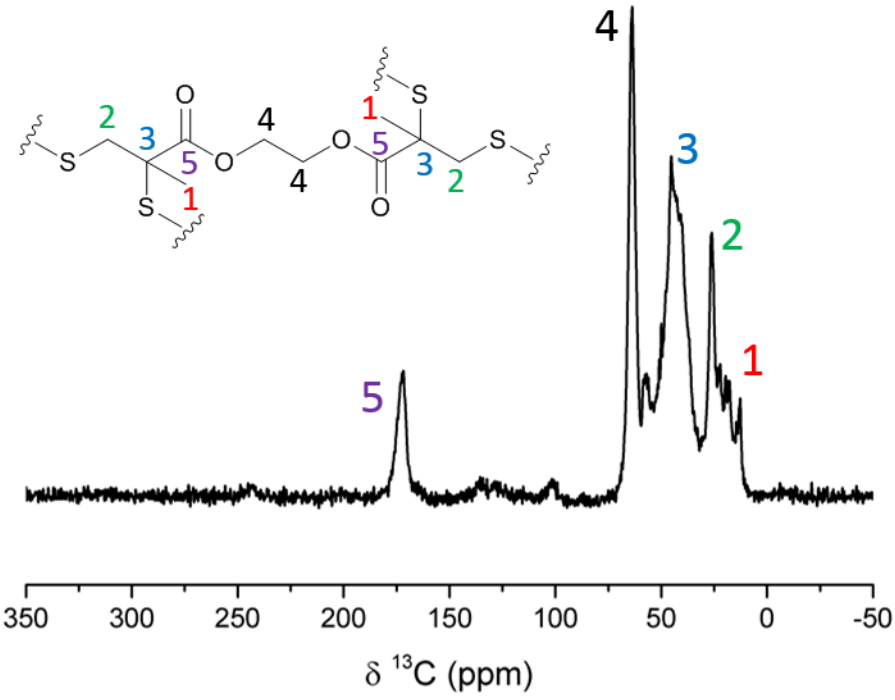

Supplementary figure 3. Solid state ^{13}C NMR spectra of ethylene glycol dimethylacrylate (EGDMA) after polymerisation with sulfur. Conditions: equal mass of sulfur and crosslinker, 1 wt.% ZnD_2 catalyst, 135 °C. The spectrum shows the formation of C-S bonds, and near complete loss of C=C signal.

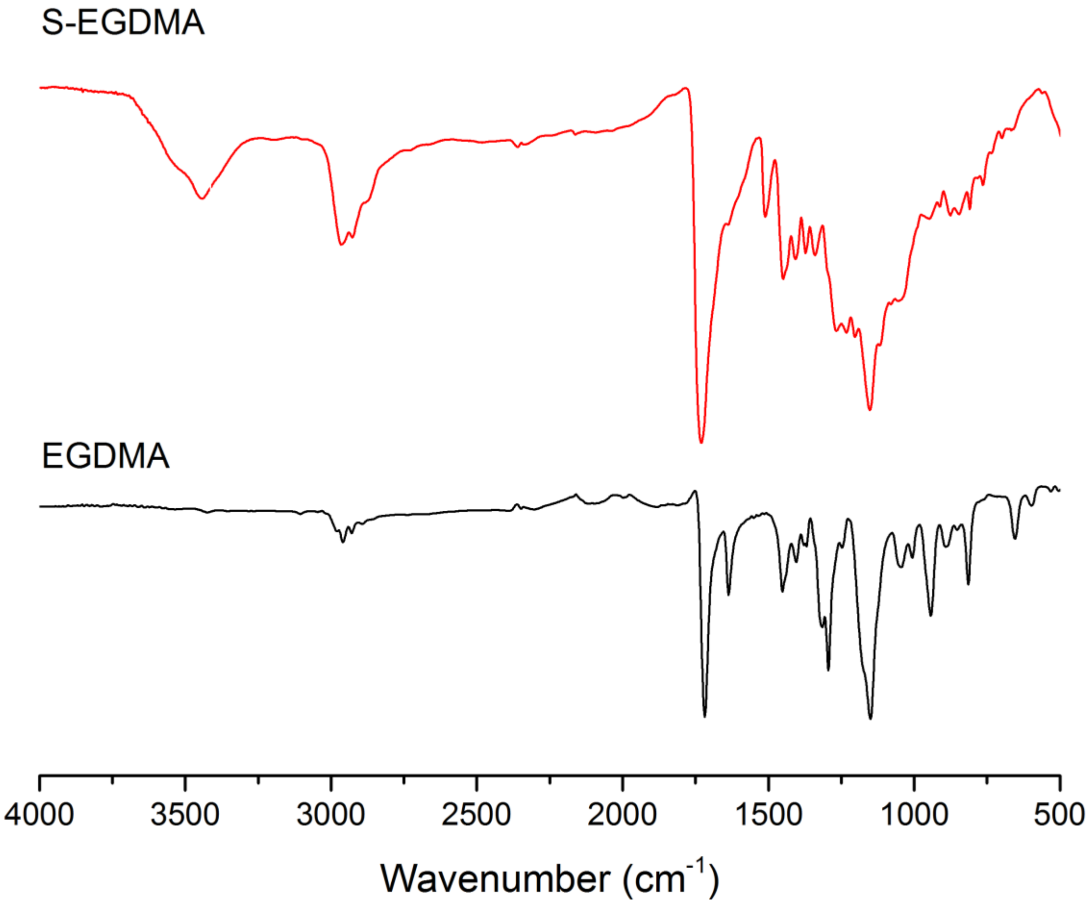

Supplementary figure 4. FT-IR spectra of EGDMA monomer, bottom, and after polymerization with sulfur, top. The reaction was carried out at a 1:1 mass ratio of sulfur to crosslinker, 5 wt.% ZnD_2 catalyst loading, at 135 °C. After polymerization there is a reduction in the signal at ~1650 cm^{-1} of the C=C stretching vibrations. There is also a reduction, in the fingerprint region, of the alkene C-H bending modes at ~650, 800, 950 cm^{-1}.

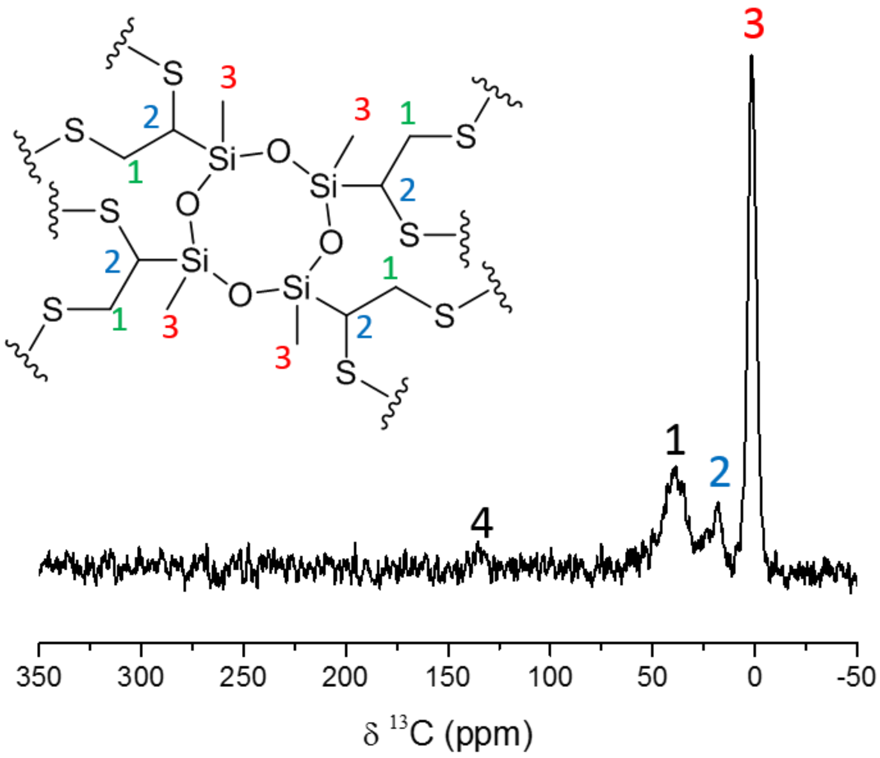

Supplementary figure 5. Solid state ^{13}C NMR spectra of 1,3,5,7-tetravinyltetramethylcyclotetrasiloxane (TVTCSi) after polymerisation with sulfur. Conditions: equal mass of sulfur and crosslinker, 1 wt.% ZnD2 catalyst, 135 °C. The spectra shows the formation of C-S bonds, and almost complete loss of C=C signals (**4**).

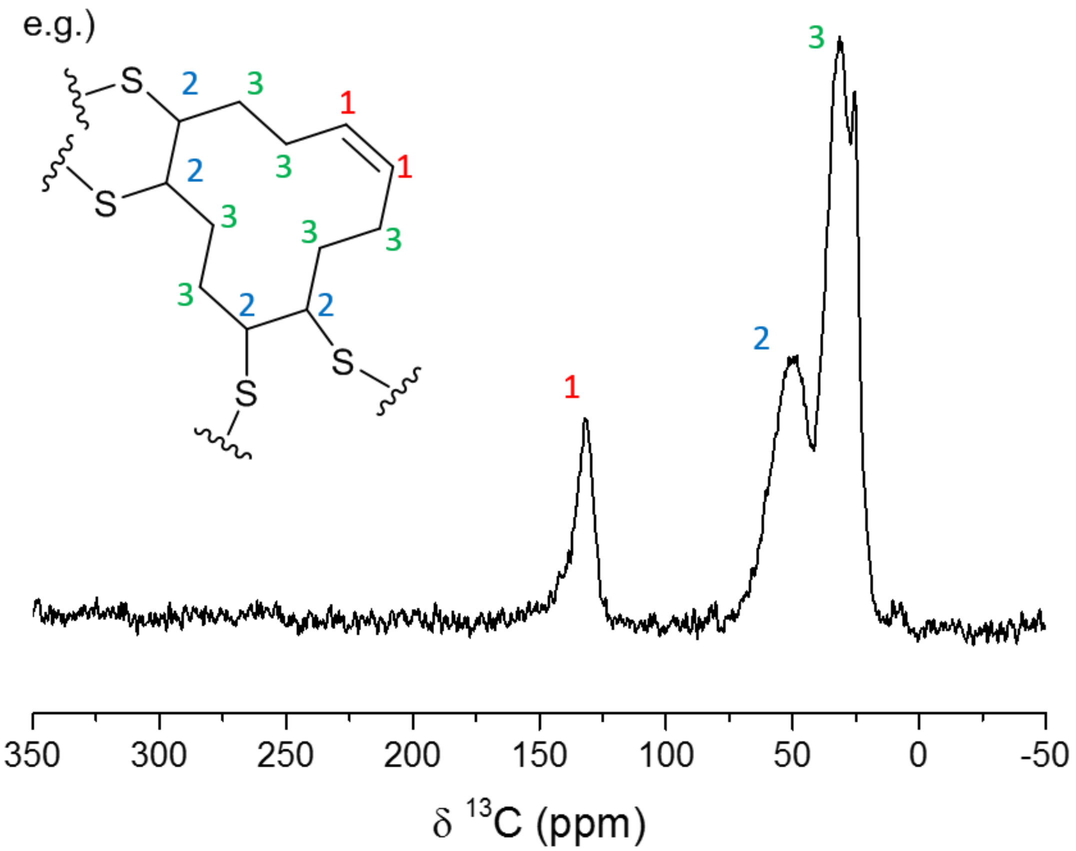

Supplementary figure 6. Solid state ^{13}C NMR spectra of 1,5,9-Cyclododecatriene (CDDT) after polymerisation with sulfur. Conditions: equal mass of sulfur and crosslinker, 1 wt.% ZnD_2 catalyst, 135 °C. The spectra shows the formation of C-S bonds, but incomplete addition across the double bonds.

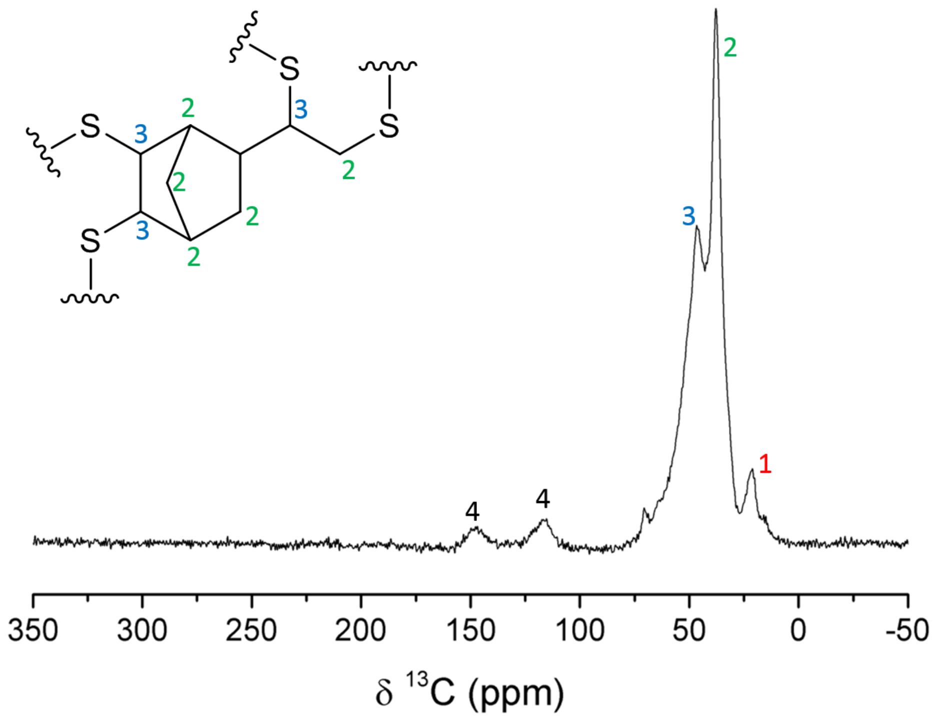

Supplementary figure 7. Solid state ^{13}C NMR spectra of 5-vinylidene-2-norbornen (VNB) after polymerisation with sulfur. Conditions: equal mass of sulfur and crosslinker, 1 wt.% ZnD_2 catalyst, 135 °C. The spectrum shows the formation of C-S bonds, and significant loss of C=C signal (**4**). This signal at position (**1**) arises from the –CH_3 moiety that results from a small proportion of VNB isomerizing to 5-ethylidene-2-norbornene.

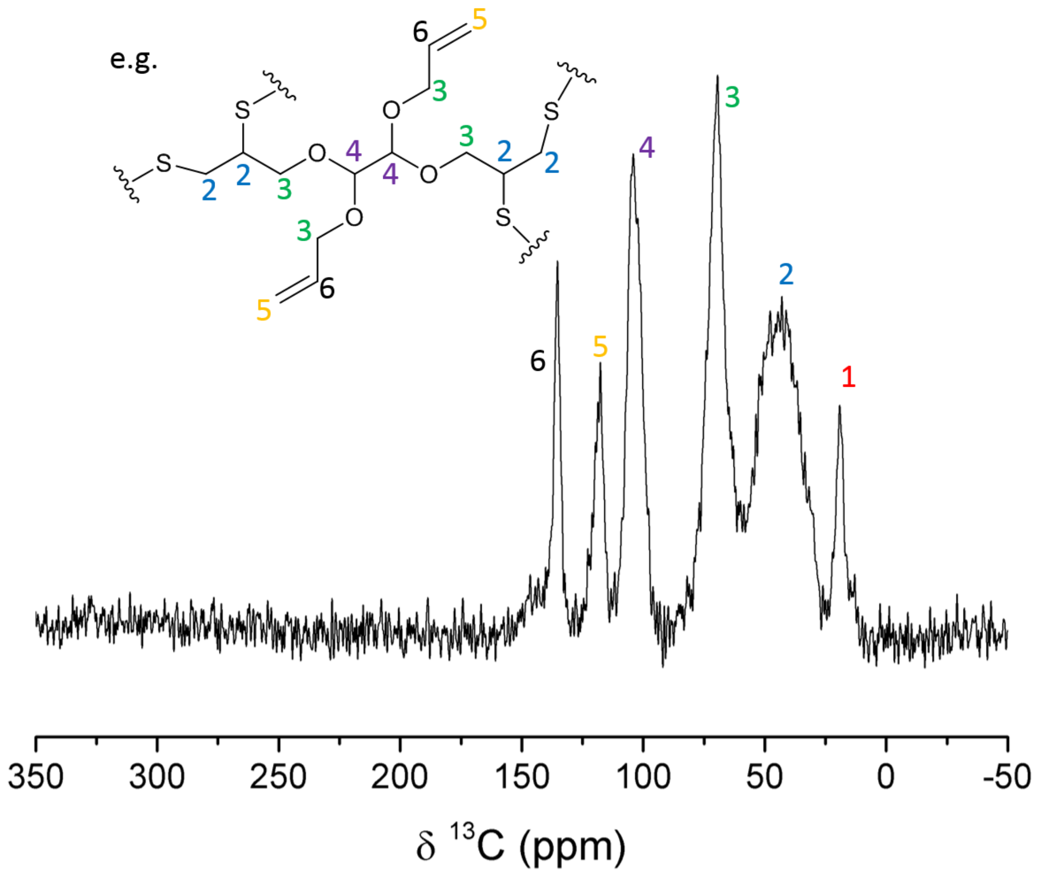

Supplementary figure 8. Solid state ^{13}C NMR spectra of glyoxal bis(diallylacetal) (GBDA) after polymerisation with sulfur. Conditions: equal mass of sulfur and crosslinker, 1 wt.% ZnD_2 catalyst, 135 °C. The spectrum shows the formation of C-S bonds, though still some significant C=C signals still present – indicative of the relatively low reactivity of this crosslinker that for many of the polymerized crosslinkers fewer than all four double bond positions may have reacted. This cause of the signal at position (**1**) is not immediately apparent, as nothing in the structure of the crosslinker or polymer should account for this. We hypothesize that some degree of isomerization, shifting the double bond position, or proton migration during reaction, may generate primary alkyl groups.

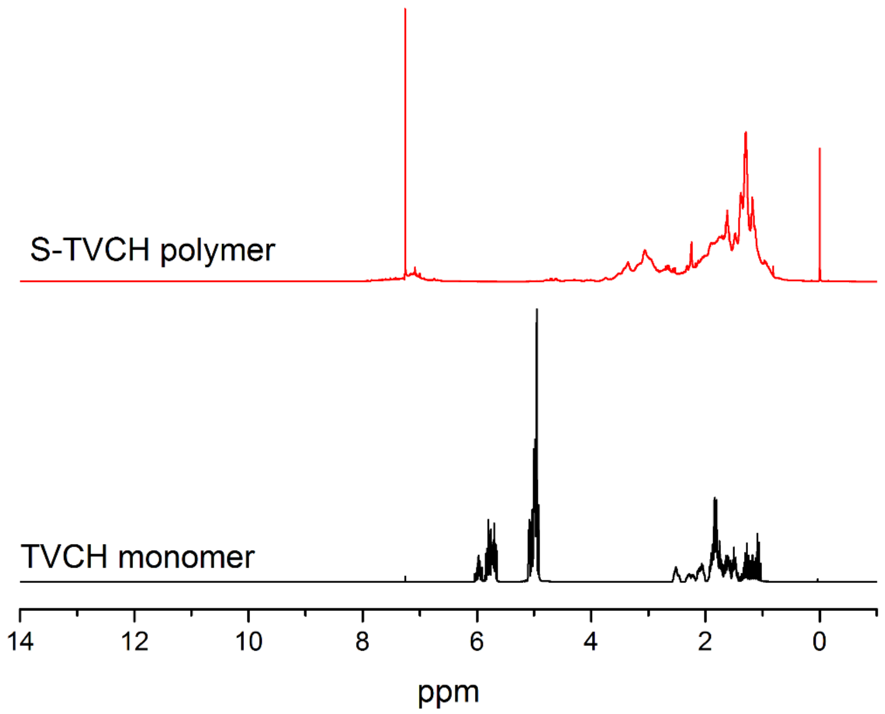

Supplementary figure 9. ^{1}H NMR spectra of 1,2,4-Trivinyl-Cyclohexane (TVCH), and after polymerization with sulfur. Polymerization with ZnD_2 as catalysts produced a material sparingly soluble in chloroform. NMR confirms conversion of all TVCH crosslinker. The NMR shown that there was no C=C double bonds remaining. The solubility of these materials may provide some very unique applications by allowing solution process-ability. For example, casting or coating of the materials. New peaks appearing in the 3-4 ppm range are indicative of H-C-S protons. It is likely that the close proximity of two of the alkene positions (i.e. the 3 and 4 substituted positions of 1,3,4 trivinyl cyclohexane), lead to a significant amount of intramolecular rather than intermolecular crosslinking – hence increasing the solubility.

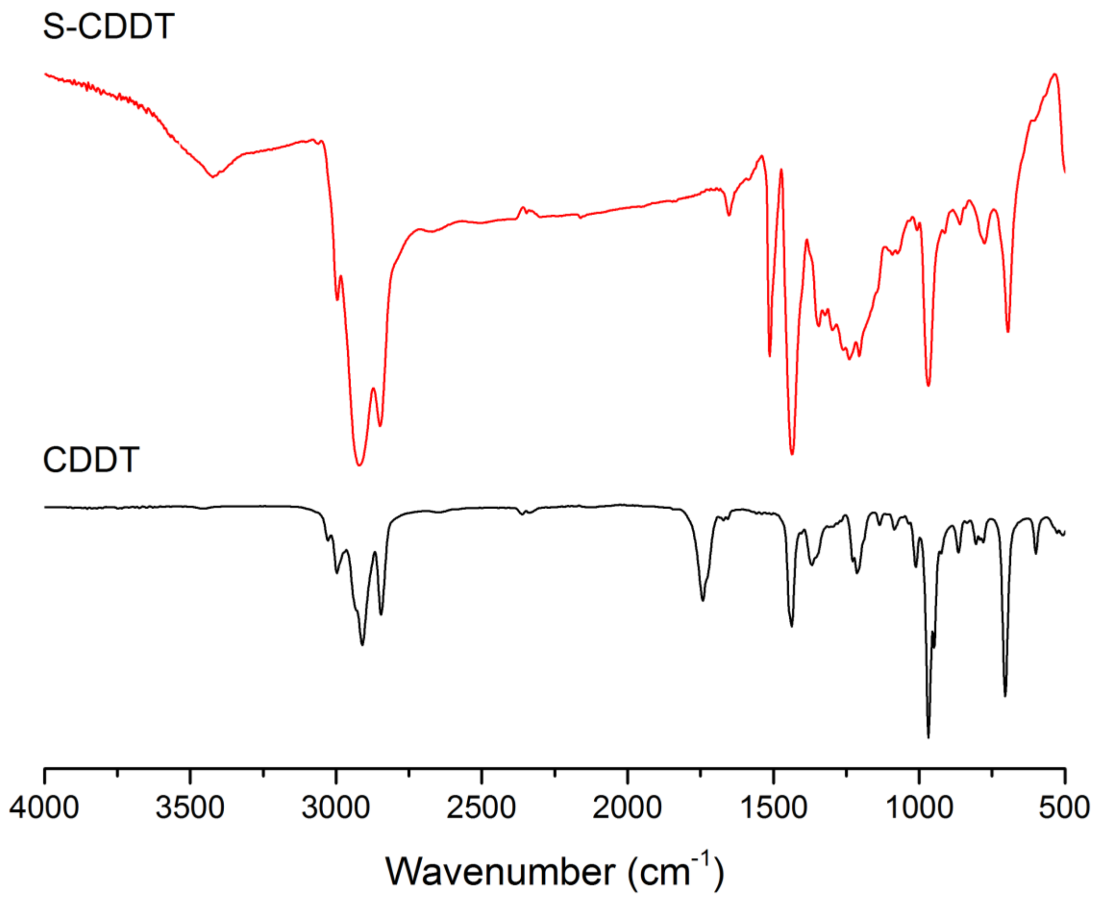

Supplementary figure 10. FT-IR spectra of CDDT monomer, bottom, and after polymerization with sulfur, top. The reaction was carried out at a 1:1 mass ratio of sulfur to crosslinker, 5 wt.% ZnD_2 catalyst loading, at 135 °C. After polymerization there is a reduction in the signals at ~3050 and ~1750 cm^{-1}, of the C=C–H and C=C stretching vibrations. There is also a reduction, in the fingerprint region, of the alkene C-H bending modes at ~600, 850, 950 cm^{-1}.

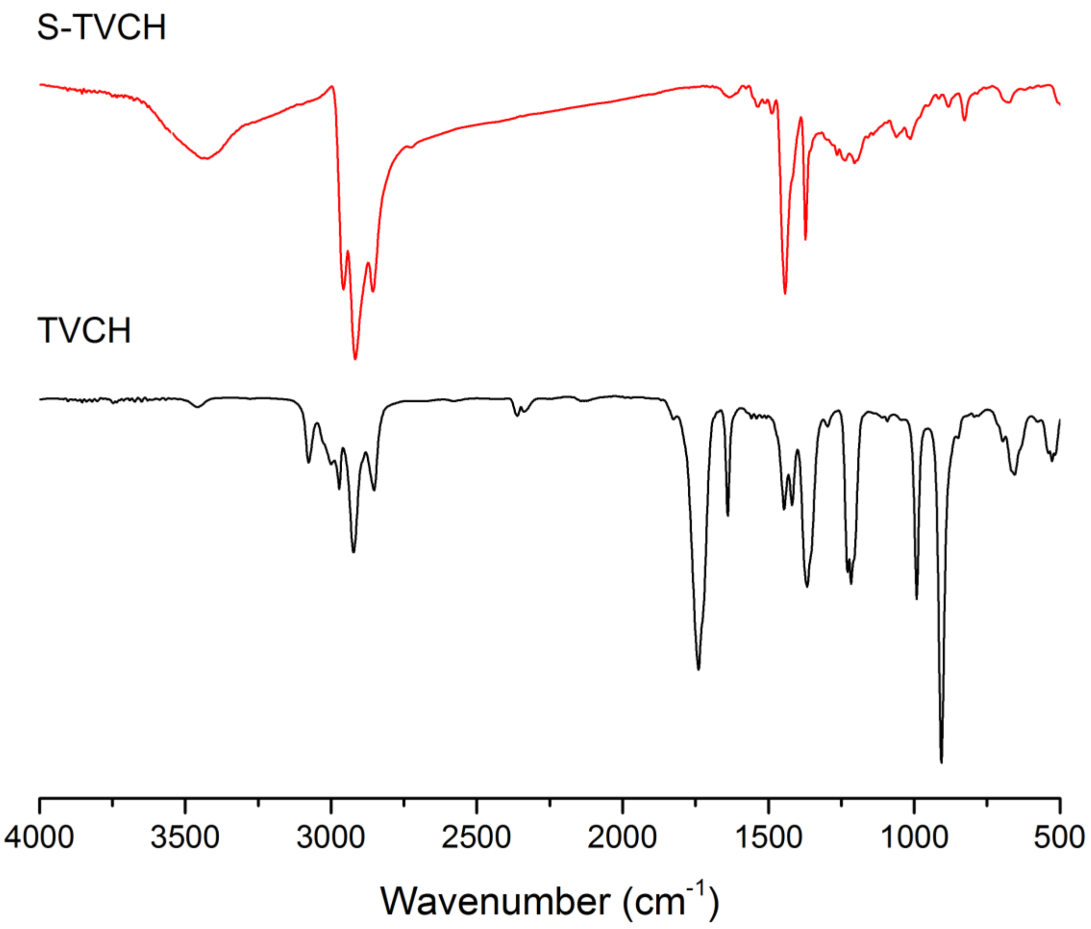

Supplementary figure 11. FT-IR spectra of TVCH monomer, bottom, and after polymerization with sulfur, top. The reaction was carried out at a 1:1 mass ratio of sulfur to crosslinker, 5 wt.% ZnD_2 catalyst loading, at 135 °C. After polymerization there is a significant reduction in the signals at ~3100 cm^{-1}, of the C=C–H vibrations, as well as at ~1750 and 1650 cm^{-1}, of the two inequivalent C=C stretching positions. There is also a significant reduction, in the fingerprint region, of the alkene C-H bending modes at ~650, 900, 1000 cm^{-1}. The almost complete disappearance of these positions suggests a very high level of conversion.

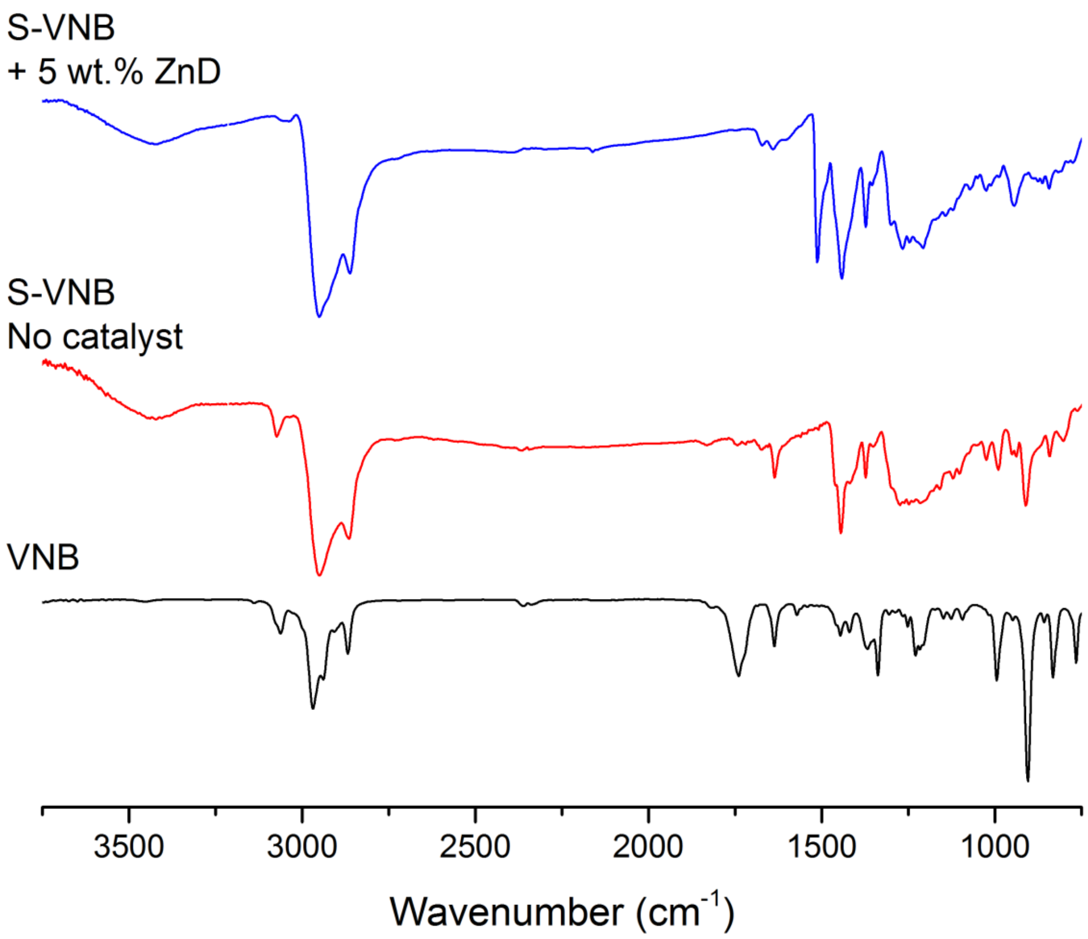

Supplementary figure 12. FT-IR spectra of VNB monomer, bottom, and after polymerization with sulfur, top. The reaction was carried out at a 1:1 mass ratio of sulfur to crosslinker, at 135 °C. After polymerization without catalyst there is a reduction in the signals at ~3100 cm^{-1}, of the C=C–H vibrations, as well as at ~1750 and 1650 cm^{-1}, of the two inequivalent C=C stretching positions. It can be noted that there is still some remaining double bond character, and that one of the positions appears to react more readily than the other. There is also a reduction, in the fingerprint region, of the alkene C-H bending modes at ~750, 900, 1000 cm^{-1}. When ZnD_2 catalyst is used there is almost complete conversion of both double bonds.

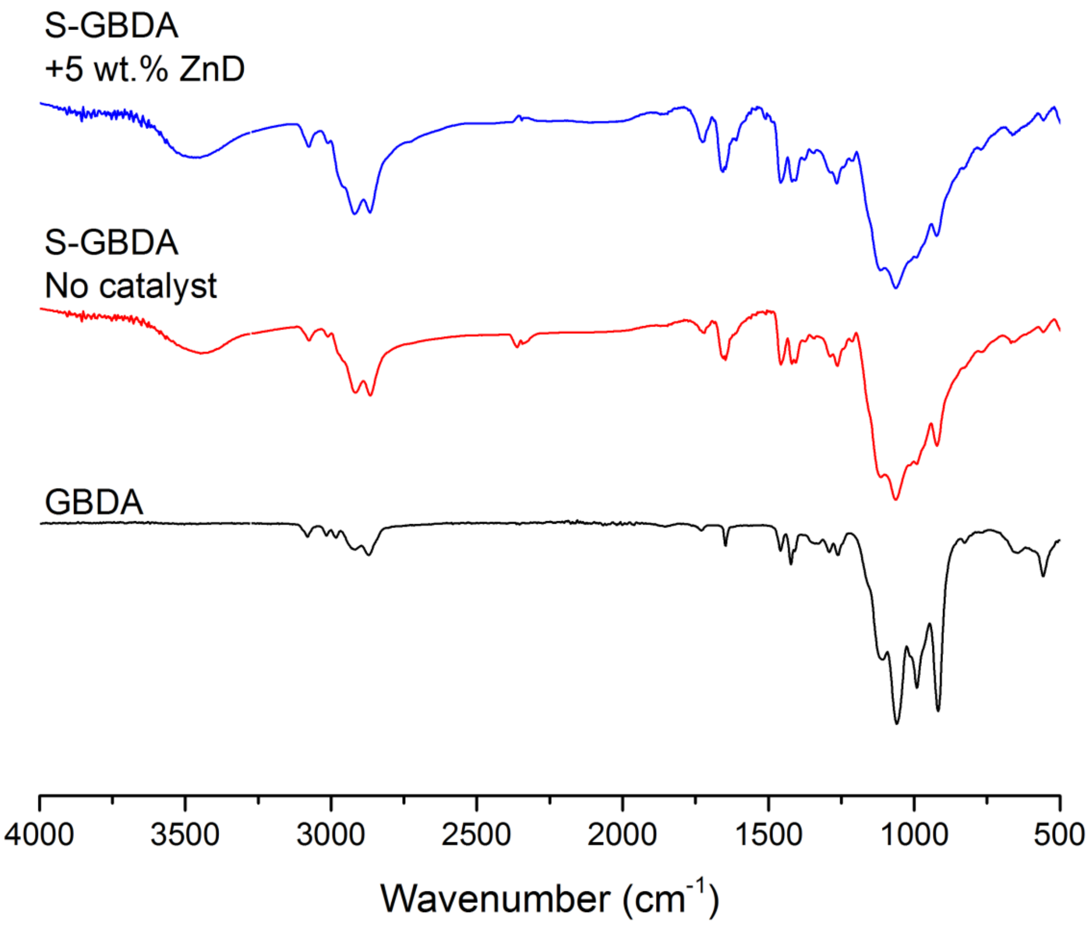

Supplementary figure 13. FT-IR spectra of GBDA monomer, bottom, and after polymerization with sulfur, top. The reaction was carried out at a 1:1 mass ratio of sulfur to crosslinker, at 135 °C. After polymerization there is a slight reduction in the signal at ~3100 cm^{-1}, corresponding to C=C–H vibrations, but little if any reduction seen at ~1650 cm^{-1}, corresponding to C=C stretching. There is also a very slight reduction, in the fingerprint region, of the alkene C-H bending modes at ~550, 900, 1000 cm^{-1}. The significant remaining C=C signals indicate there has not been a complete reaction, even when catalyst is used.

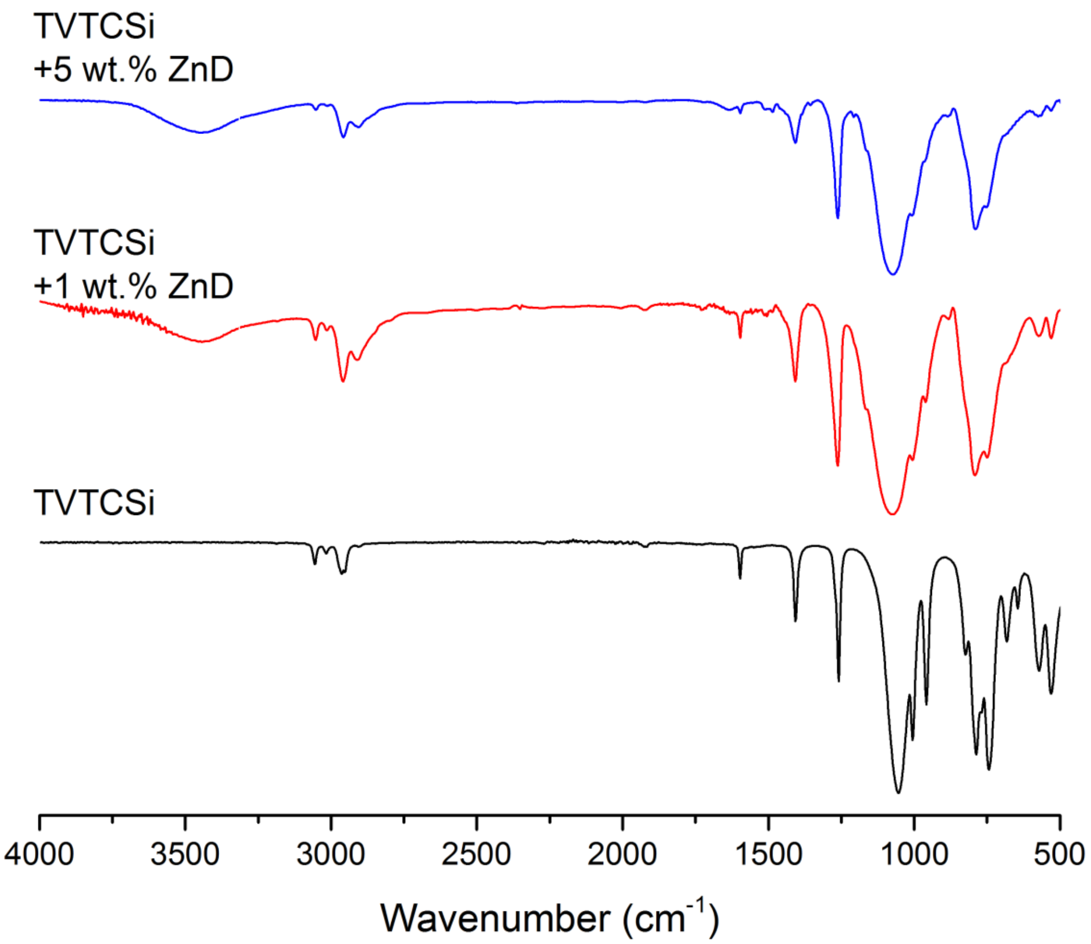

Supplementary figure 14. FT-IR spectra of TVTCSi monomer, bottom, and after polymerization with sulfur, top. The reaction was carried out at a 1:1 mass ratio of sulfur to crosslinker, at 160 °C. After polymerization with 1 wt.% ZnD_2 there is a slight reduction in the signal at ~3050 cm^{-1}, corresponding to C=C–H vibrations, but little if any reduction seen at ~1600 cm^{-1}, corresponding to C=C stretching. There is also a very slight reduction, in the fingerprint region, of the alkene C-H bending modes at ~550, 950, 1000 cm^{-1}. The significant remaining C=C signals indicate there has not been a complete reaction – perhaps unsurprising considering the relative unreactivity of this crosslinker compared to the others, requiring an elevated temperature (160 rather than 135 °C) in order to react. After polymerization with 5 wt.% catalyst there is noticeably more depletion of the C=C bonds.

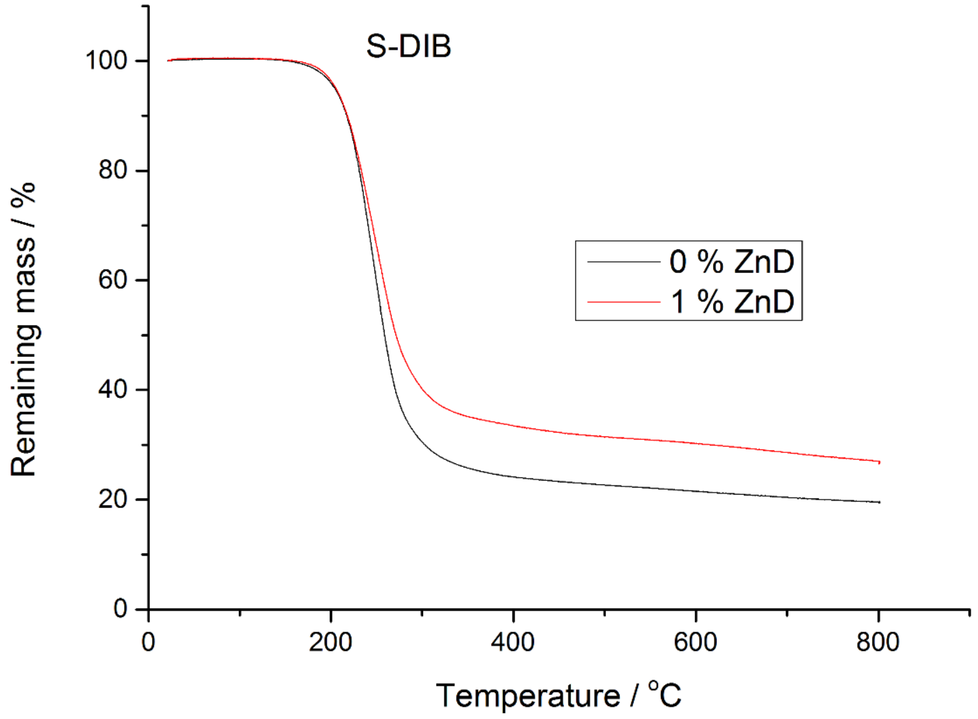

Supplementary figure 15: Thermogravimetric analysis, under nitrogen, of S-DIB copolymers (equal mass ratio of sulfur to crosslinker) with and without catalysis by Zn diethyldithiocarbamate.

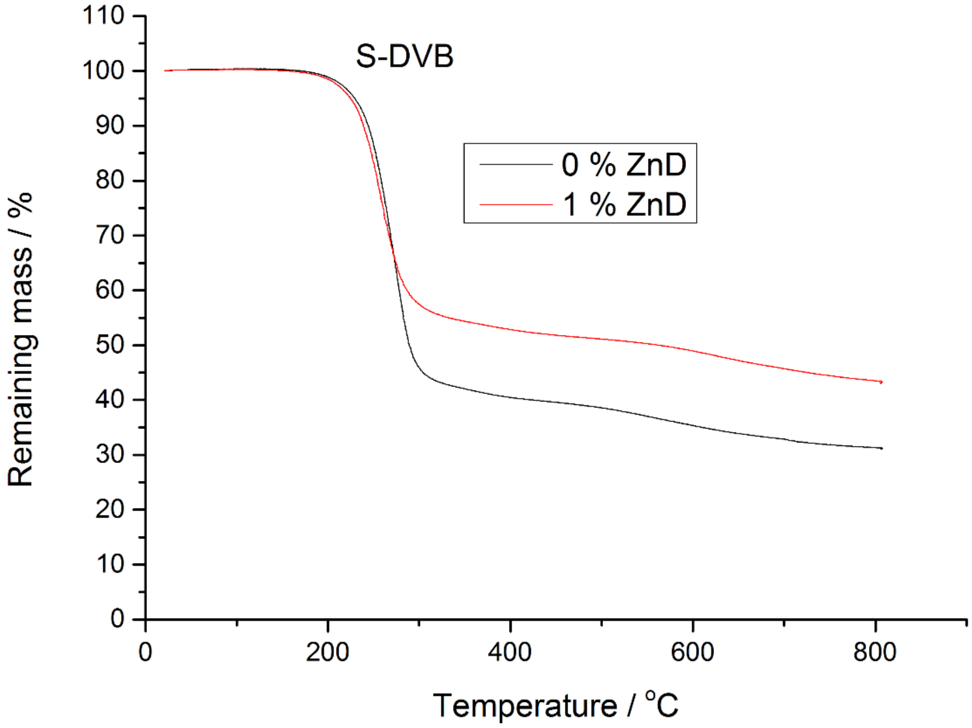

Supplementary figure 16: Thermogravimetric analysis, under nitrogen, of S-DVB copolymers (equal mass ratio of sulfur to crosslinker) with and without catalysis by Zn diethyldithiocarbamate.

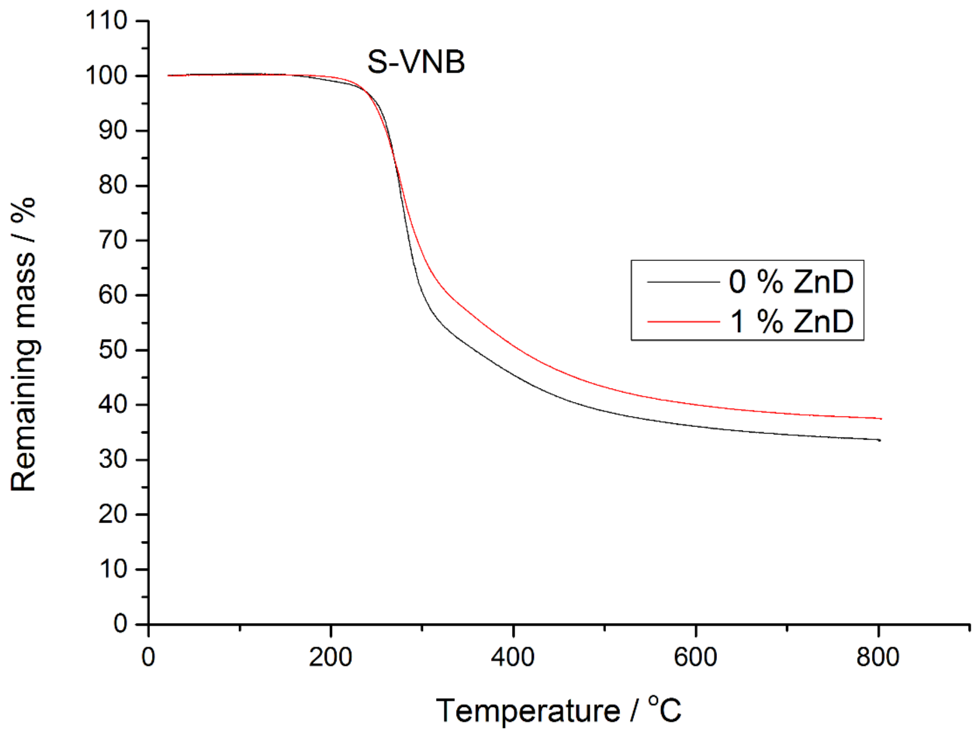

Supplementary figure 17: Thermogravimetric analysis, under nitrogen, of S-VNB copolymers (equal mass ratio of sulfur to crosslinker) with and without catalysis by Zn diethyldithiocarbamate.

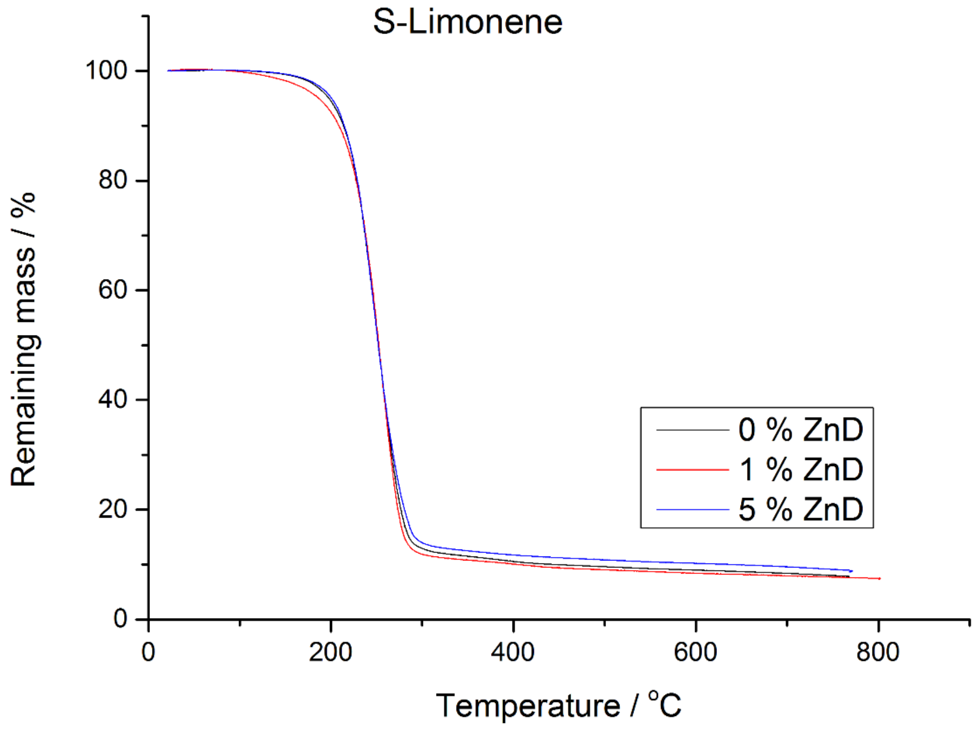

Supplementary figure 18: Thermogravimetric analysis, under nitrogen, of S-limonene copolymers (equal mass ratio of sulfur to crosslinker) with and without catalysis by Zn diethyldithiocarbamate.

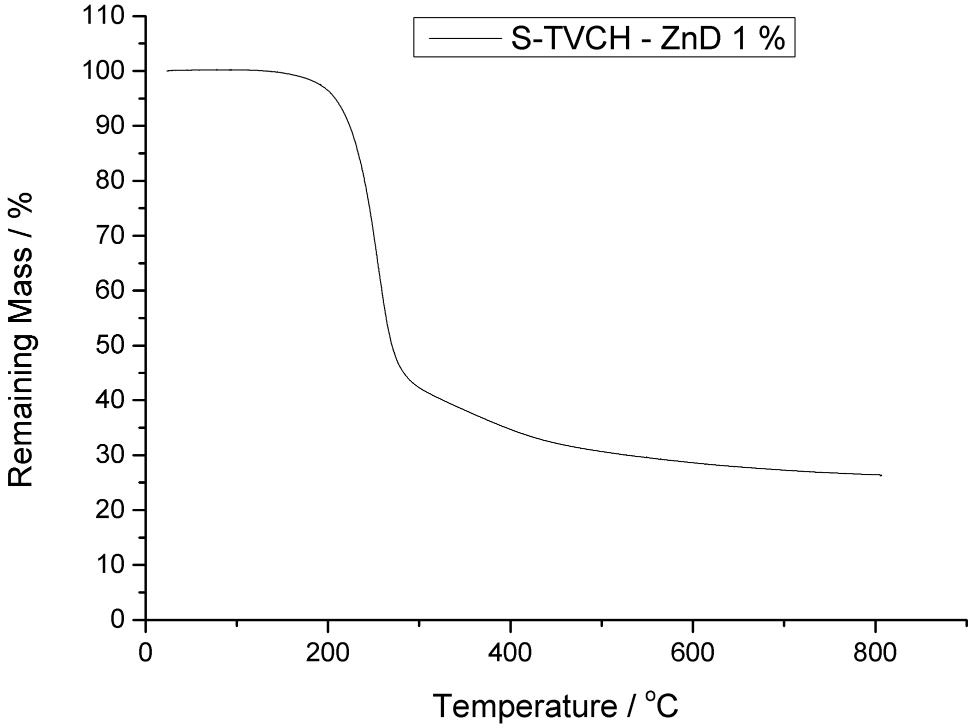

Supplementary figure 19: Thermogravimetric analysis, under nitrogen, of S-TVCH copolymer (equal mass ratio of sulfur to crosslinker) catalysed by Zn diethyldithiocarbamate.

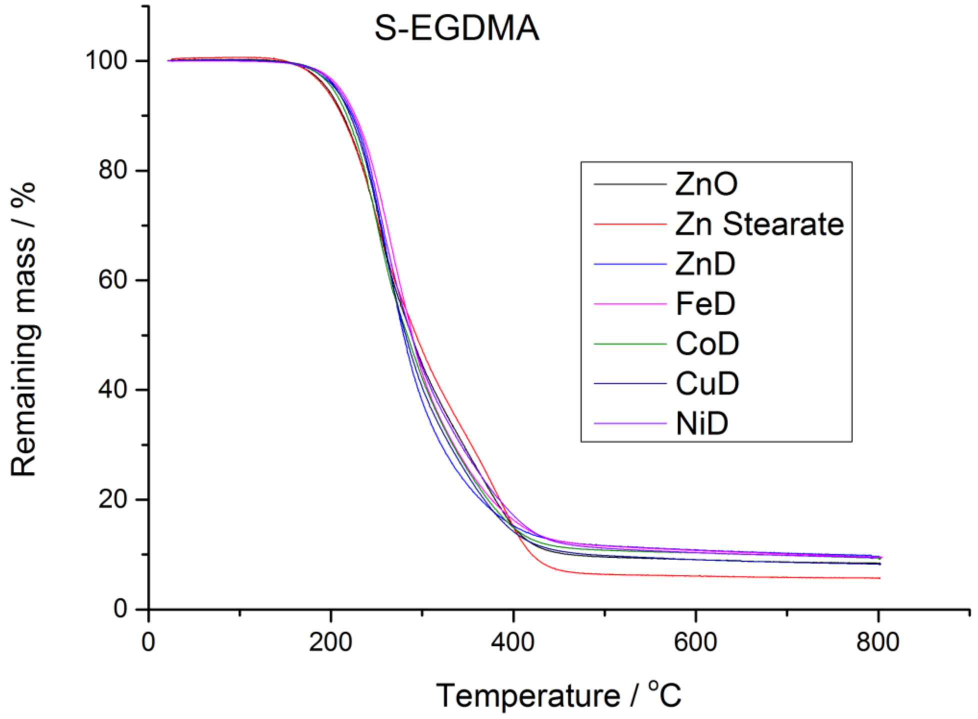

Supplementary figure 20: Thermogravimetric analysis, under nitrogen, of S-EGDMA copolymers (equal mass ratio of sulfur to crosslinker) with a range of potential catalysts, all at 1 wt.% loading.

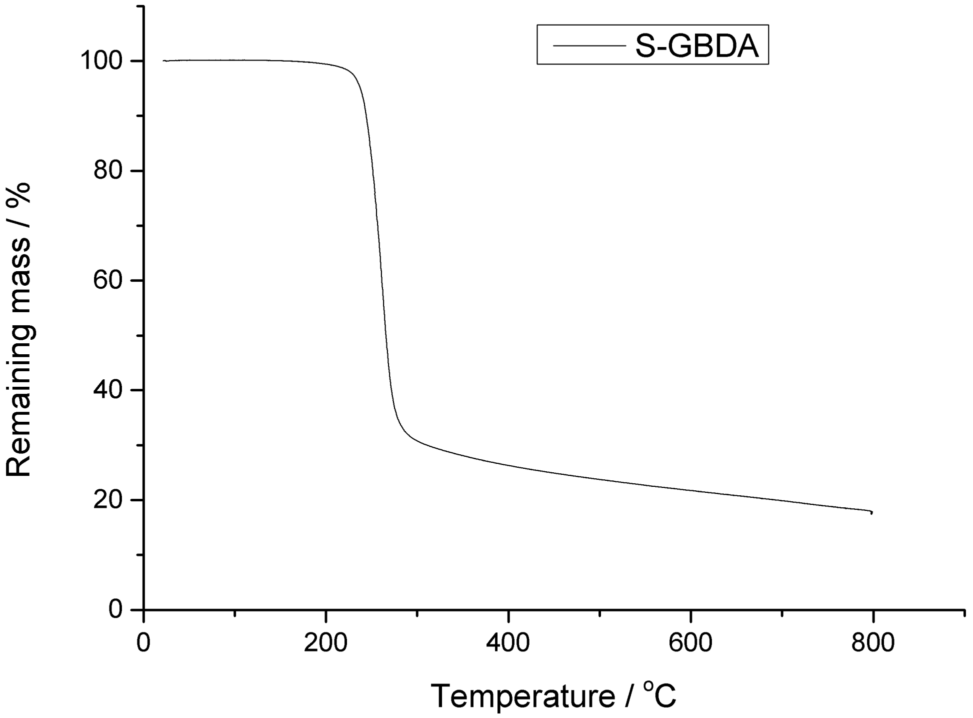

Supplementary figure 21: Thermogravimetric analysis, under nitrogen, of S-GBDA copolymer (equal mass ratio of sulfur to crosslinker) catalysed by Zn diethyldithiocarbamate.

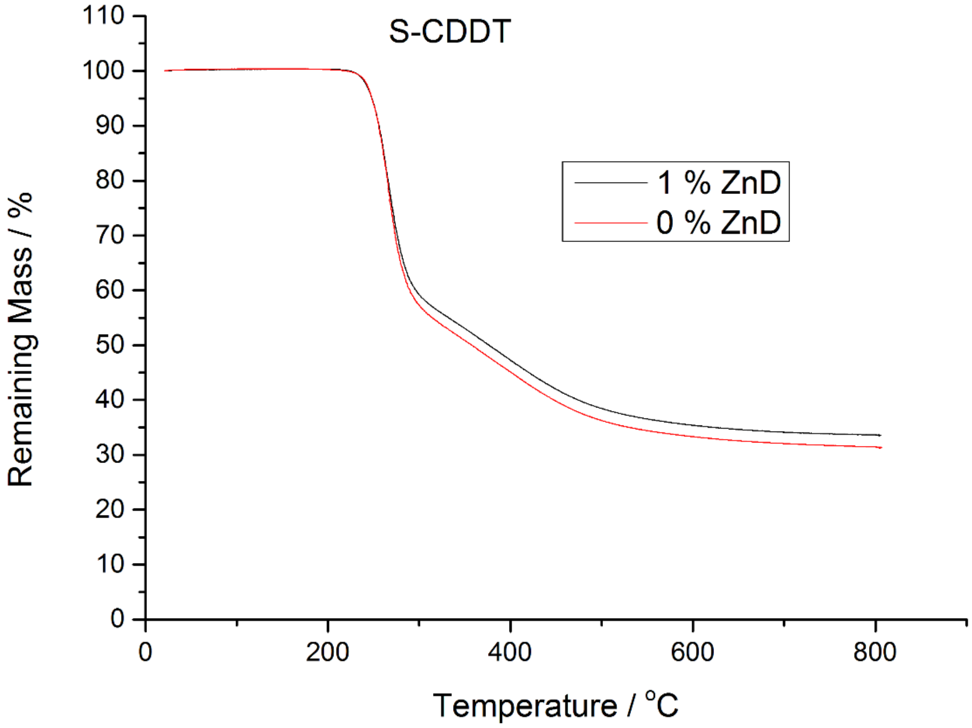

Supplementary figure 22: Thermogravimetric analysis, under nitrogen, of S-CDDT copolymers (equal mass ratio of sulfur to crosslinker) with and without catalysis by Zn diethyldithiocarbamate.

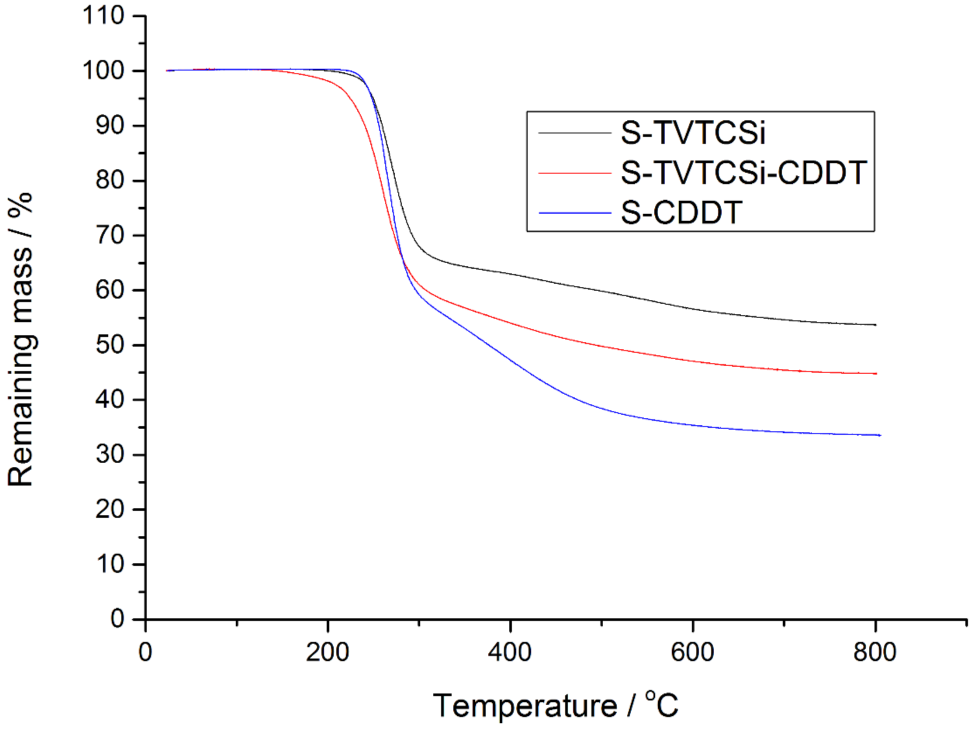

Supplementary figure 23: Thermogravimetric analysis, under nitrogen, of S-crosslinker copolymers (equal mass ratio of sulfur to crosslinker) catalysed by Zn diethyldithiocarbamate (1 wt.%). The crosslinkers used were either TVTCSi, CDDT, or an equal weight mixture of the two. Blending of the crosslinkers gives an average of the two residual masses.

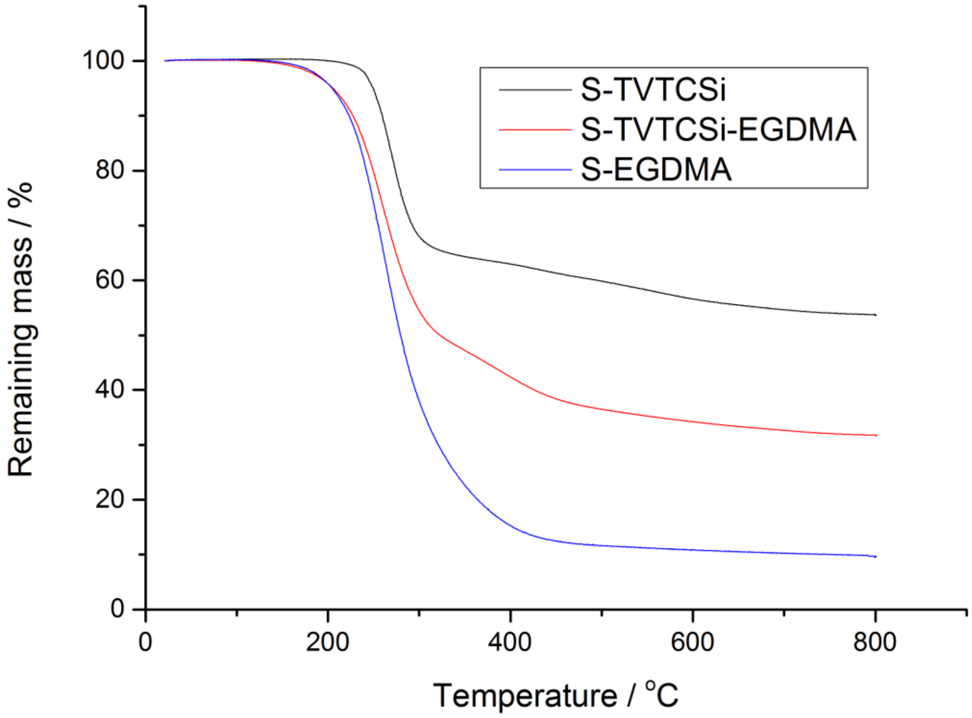

Supplementary figure 24: Thermogravimetric analysis, under nitrogen, of S-crosslinker copolymers (equal mass ratio of sulfur to crosslinker) catalysed by Zn diethyldithiocarbamate (1 wt.%). The crosslinkers used were either TVTCSi, EGDMA, or an equal weight mixture of the two. Blending of the crosslinkers gives an average of the two residual masses.

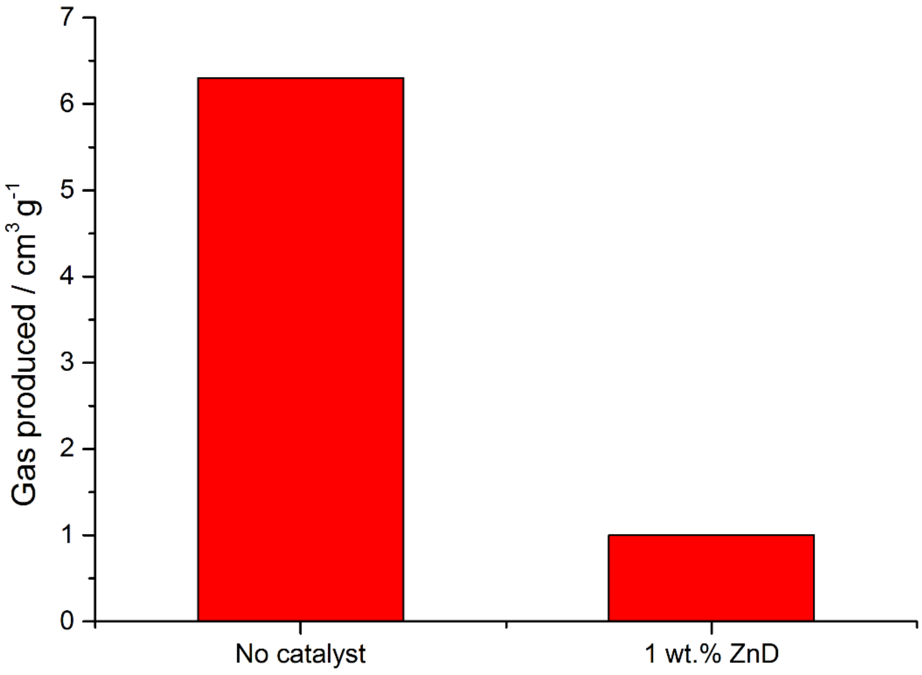

Supplementary figure 25. Volume of gas produced during reactions of sulfur with limonene, with and without catalyst. Both reactions were at an equal mass ratio of sulfur to crosslinker, at 180 °C.

Supplementary table 4 – Blending crosslinkers with sulfur. Equal weight ratio of sulfur to crosslinker, and equal weight ratio of the two crosslinkers. Blending different crosslinkers with TVTCSi can produce sSupplementary table polymers ranging from glassy solids with no detecSupplementary table T_g (+CDDT and +DCPD), to rubbery solids with sub-room temperature T_g (+EGDMA, 8.7 °C; +Farnesol, 4.3 °C, Supplementary table 4).

Entry	Cross-linkers	Tempt / °C	Description	Tg
6	TVTCSi/CDDT	135	Black solid	None detected
7	TVTCSi/DCPD	135	Black solid	None detected
8	TMTVCSi/EGDMA	135	Black solid	8.7 °C
9	TMTVCSi/Farnesol	135	Dark red solid	4.3 °C

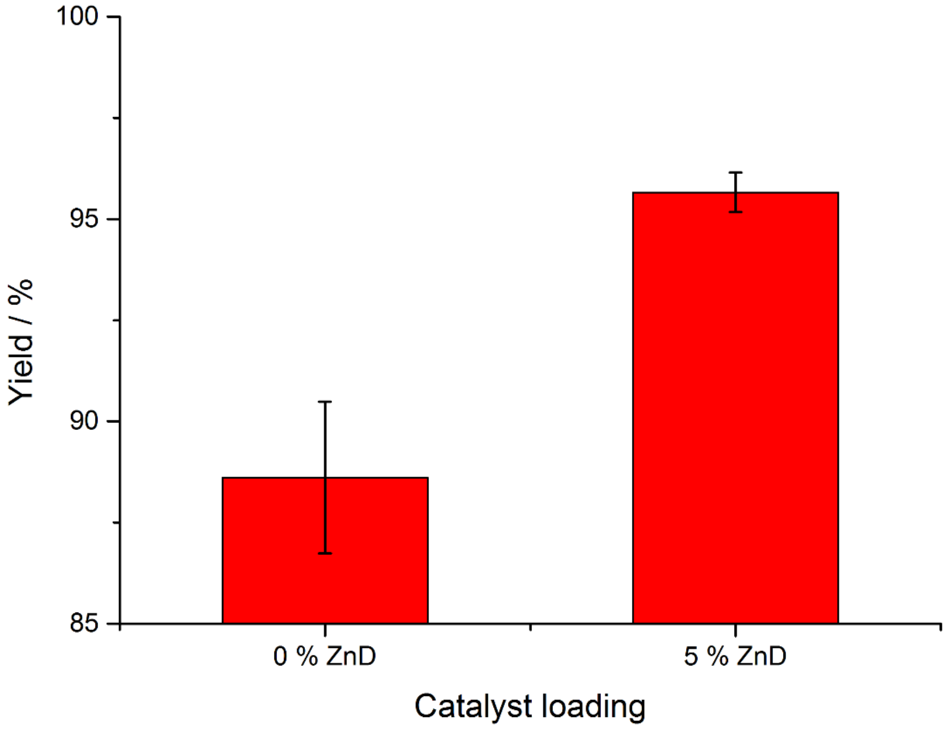

Supplementary figure 26. Investigation of the effect of catalyst on reaction yield at higher temperatures (in comparison to Supplementary figure 2f of the main paper, performed at 135 °C. The crosslinker used was DCPD, and the crosslinker to sulfur mass ratio was 1:1 (5 g each), reacted at 160 °C with stirring in a 40 mL open glass vial, for 12 hours. This shows catalysis also provides a benefit to yield in the high temperature regime.

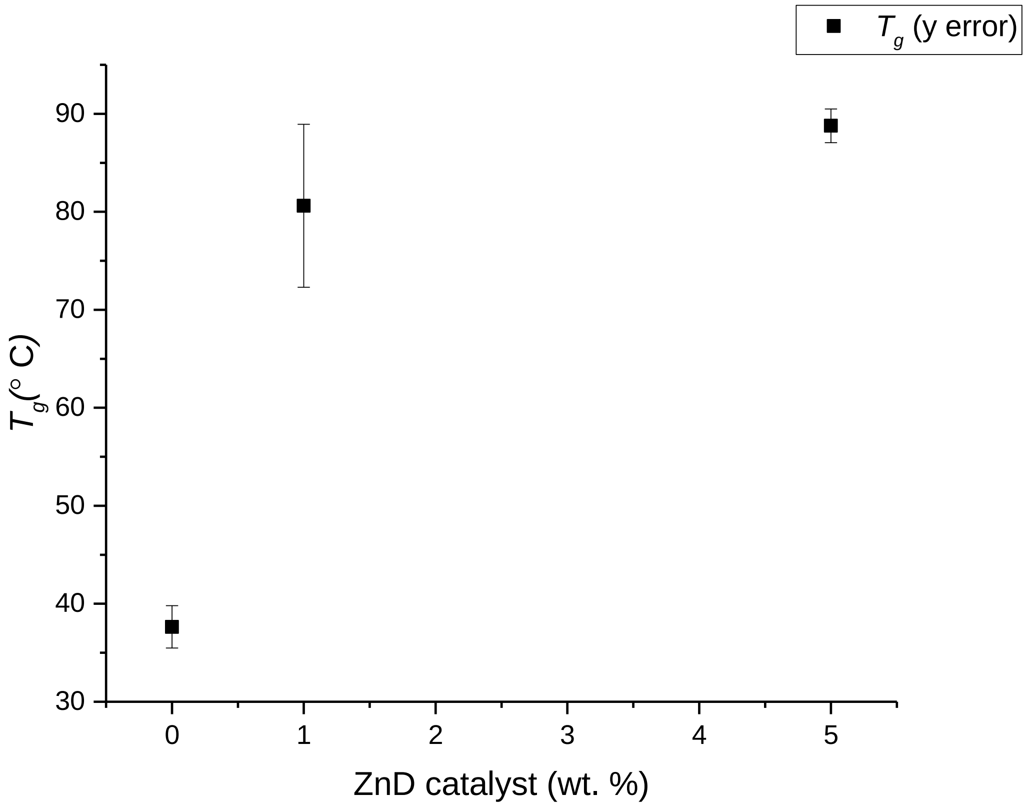

Supplementary figure 27. Glass transition temperatures (from DSC) as a function of catalyst loading for the polymerization of sulfur with DCPD. The reaction was carried out at a 1:1 mass ratio of sulfur to crosslinker, without catalyst, at 135 °C, and in triplicate. Standard deviation is given for the average of three parallel reactions. There is a significant increase in glass transition temperature for the 1 % catalyzed sample in comparison to the uncatalyzed sample. There is then a slight, though reduced increase when the catalyst loading is increased to 5 %.

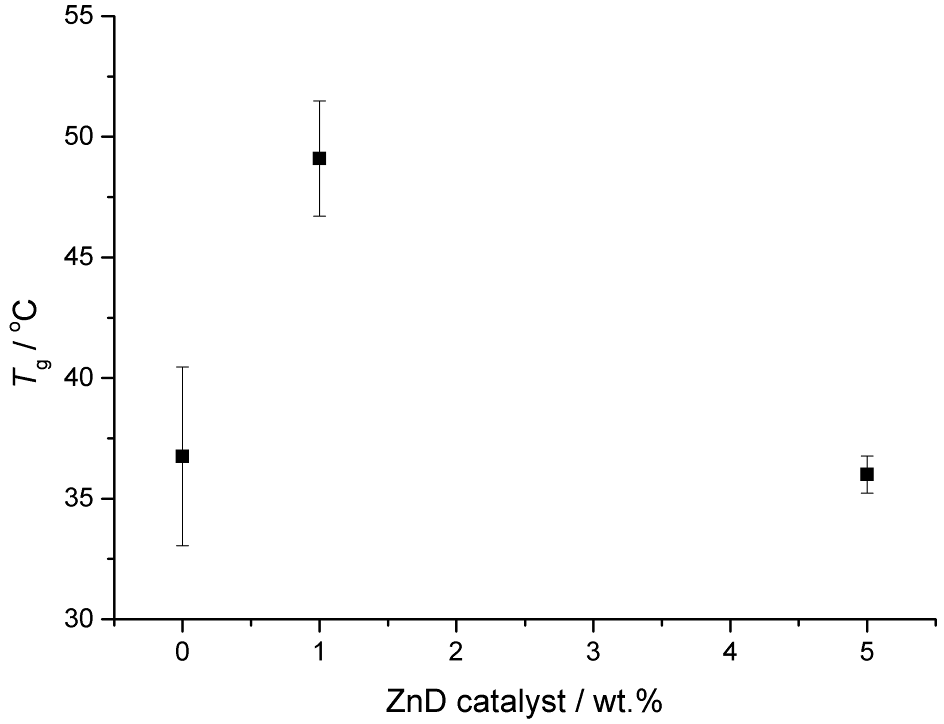

Supplementary figure 28. Glass transition temperatures (from DSC) as a function of catalyst loading for the polymerization of sulfur with DIB. The reaction was carried out at a 1:1 mass ratio of sulfur to crosslinker, without catalyst, at 135 °C, and in triplicate. Standard deviation is given for the average of three parallel reactions. There is an increase in glass transition temperature for the 1 % catalyzed sample in comparison to the uncatalyzed sample. There is then a reduction when the catalyst loading is increased to 5 %, presumably as this is then effectively an excess loading of catalyst, beyond which the catalyst is not providing additional benefit to the reaction, but is plasticising the material, as a small molecular additive.

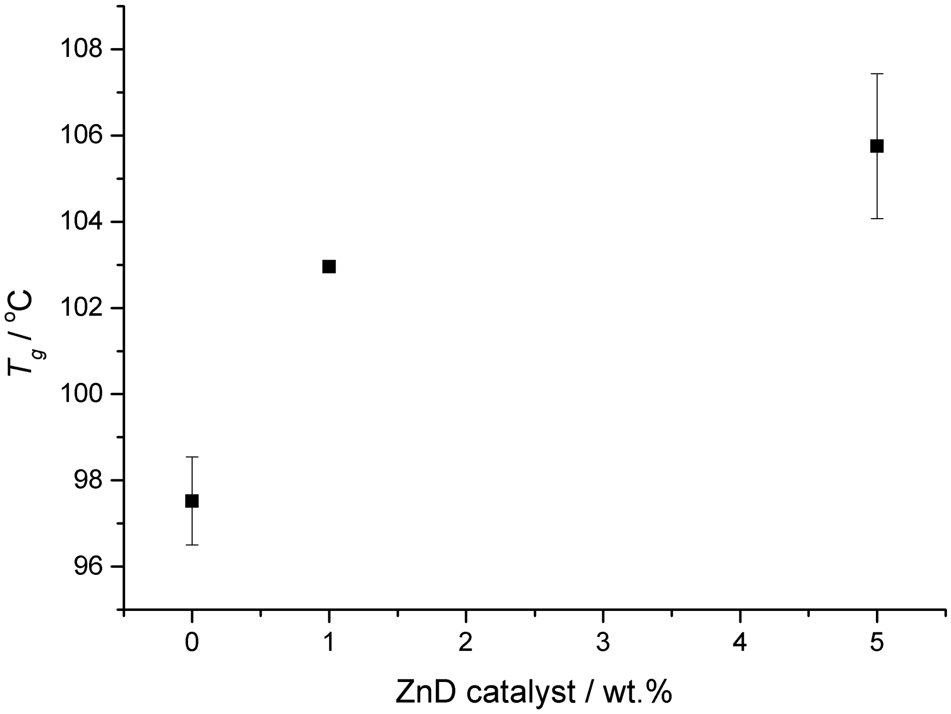

Supplementary figure 29. Glass transition temperatures (from DSC) as a function of catalyst loading for the polymerization of sulfur with DVB. The reaction was carried out at a 1:1 mass ratio of sulfur to crosslinker, without catalyst, at 135 °C, and in triplicate. Standard deviation is given for the average of three parallel reactions. There is a significant increase in glass transition temperature for the 1 % catalyzed sample in comparison to the uncatalyzed sample. There is then a slight, though reduced increase when the catalyst loading is increased to 5 %.

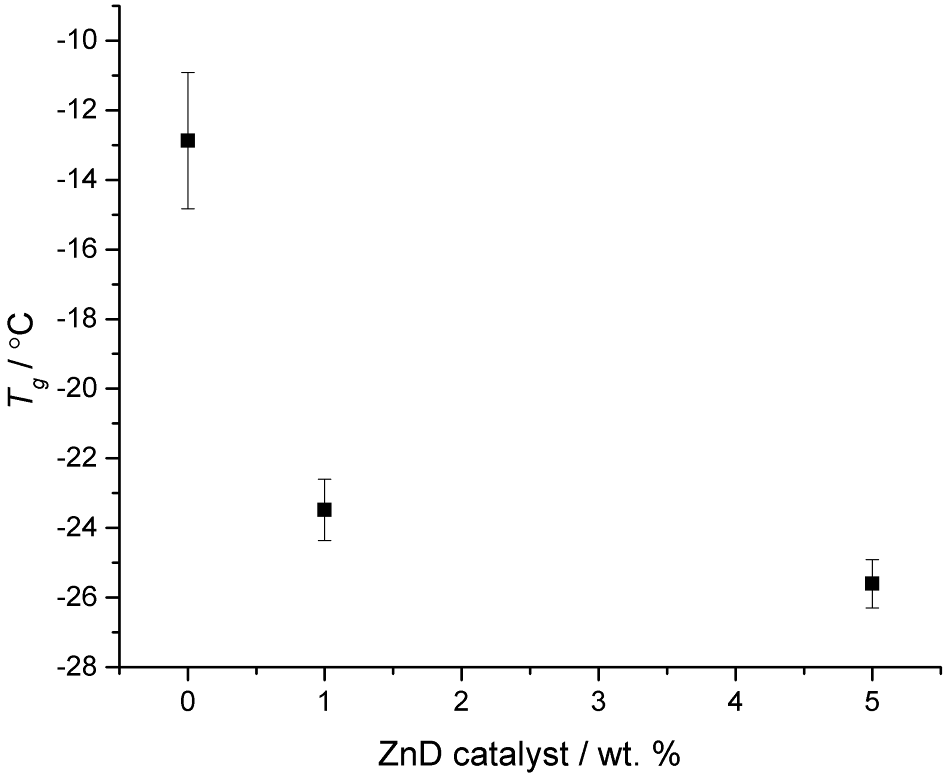

Supplementary figure 30. Glass transition temperatures (from DSC) as a function of catalyst loading for the polymerization of sulfur with linseed oil. The reaction was carried out at a 1:1 mass ratio of sulfur to crosslinker, without catalyst, at 135 °C, and in triplicate. Standard deviation is given for the average of three parallel reactions. There is a decrease in the T_gs of the polymers as a result of catalysis. It is not clear why this is the case, however, as linseed oil is a crude mixture of components, this may well be the result of quicker solidification of the polymer, and higher crosslinking, trapping more of the lower molecular weight components during reaction, that would otherwise be lost. These lower molecular weight components may then act to plasticise the material.

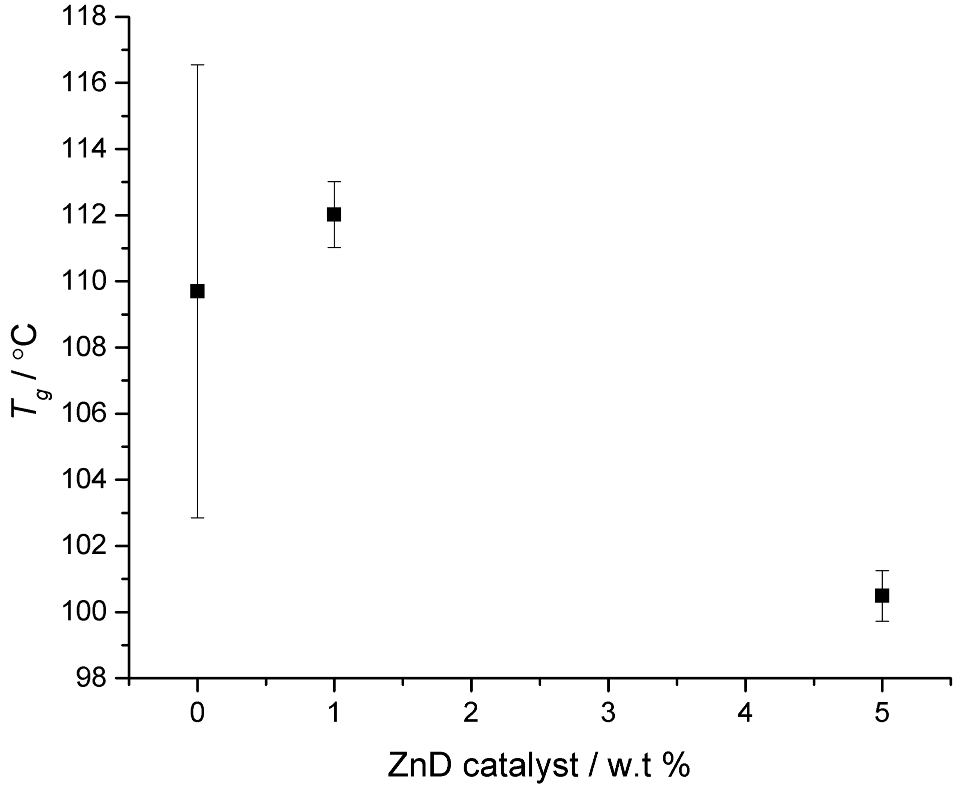

Supplementary figure 31. Glass transition temperatures (from DSC) as a function of catalyst loading for the polymerization of sulfur with VNB. The reaction was carried out at a 1:1 mass ratio of sulfur to crosslinker, without catalyst, at 135 °C, and in triplicate. Standard deviation is given for the average of three parallel reactions. There is a slight increase in glass transition temperature for the 1 % catalyzed sample in comparison to the uncatalyzed sample. There is then a significant reduction when the catalyst loading is increased to 5 %, presumably as this is then effectively an excess loading of catalyst, beyond which the catalyst is not providing additional benefit to the reaction, but is plasticising the material, as a small molecular additive. It should be noted that the T_gs for the 0 wt.% loaded samples were very faint, however the 1 and 5 wt.% catalyst loaded samples were more pronounced.

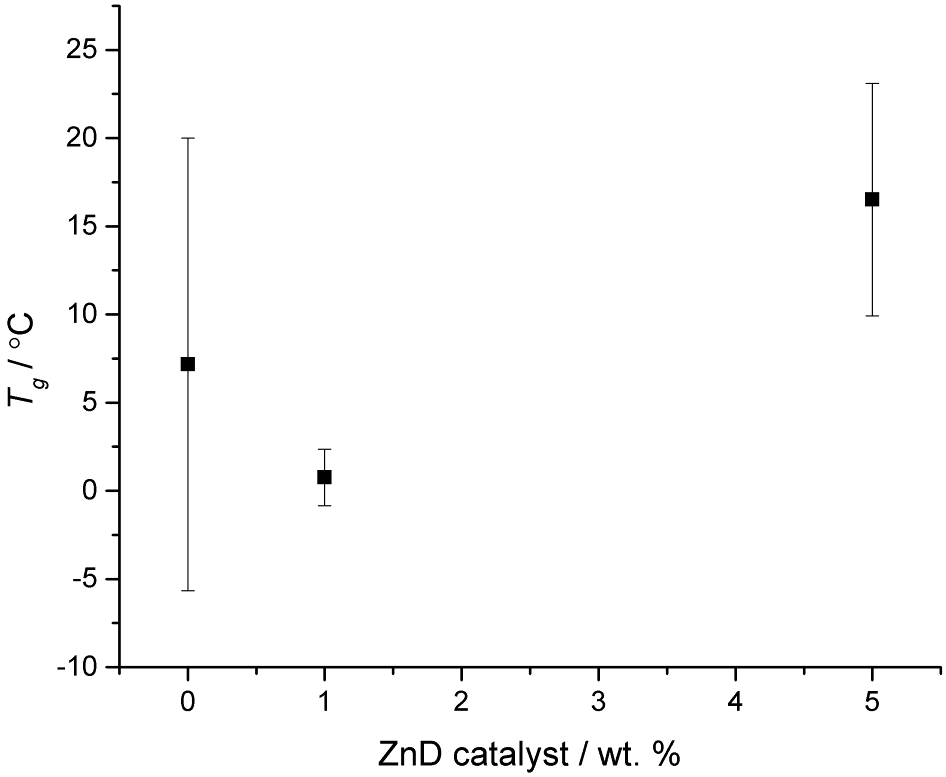

Supplementary figure 32. Glass transition temperatures (from DSC) as a function of catalyst loading for the polymerization of sulfur with GBDA. The reaction was carried out at a 1:1 mass ratio of sulfur to crosslinker, without catalyst, at 135 °C, and in triplicate. Standard deviation is given for the average of three parallel reactions. Only very faint transitions were detected, which is likely the cause of the poor accuracy in measurement. There appears to be a slight decrease in glass transition temperature moving from the zero to 1% loaded sample, and then an increase in the 5% loaded sample, however, in view of the poor reproducibility, this may not be significant.

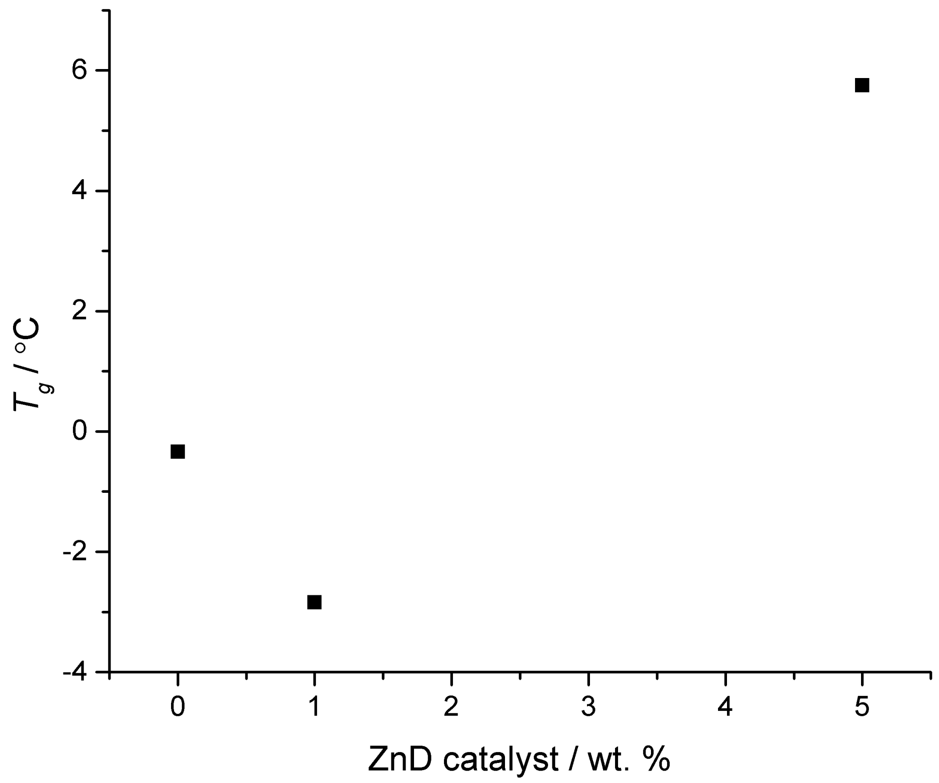

Supplementary figure 33. Glass transition temperatures (from DSC) as a function of catalyst loading for the polymerization of sulfur with limonene. The reaction was carried out at a 1:1 mass ratio of sulfur to crosslinker, according to the reported procedure. There appears to be a slight decrease in glass transition temperature moving from the zero to 1% loaded sample, and then an increase in the 5% loaded sample.

Supplementary table 5 – Glass transition temperatures for polymers where fewer than three catalyst loadings gave values. The reaction was carried out at a 1:1 mass ratio of sulfur to crosslinker, without catalyst, at 135 °C, and in triplicate. Standard deviation is given for the average of three parallel reactions.

Crosslinker	0 wt.% ZnD_2	1 wt.% ZnD_2	5 wt.% ZnD_2
TVCH	Not measured	37.1°C ±2.6	22.8°C ±1.3
CDDT	Not measured	35.1 °C ±0.9	None detected
EGDMA	No reaction	19.9 °C ±0.6	20.3 °C ±3.6

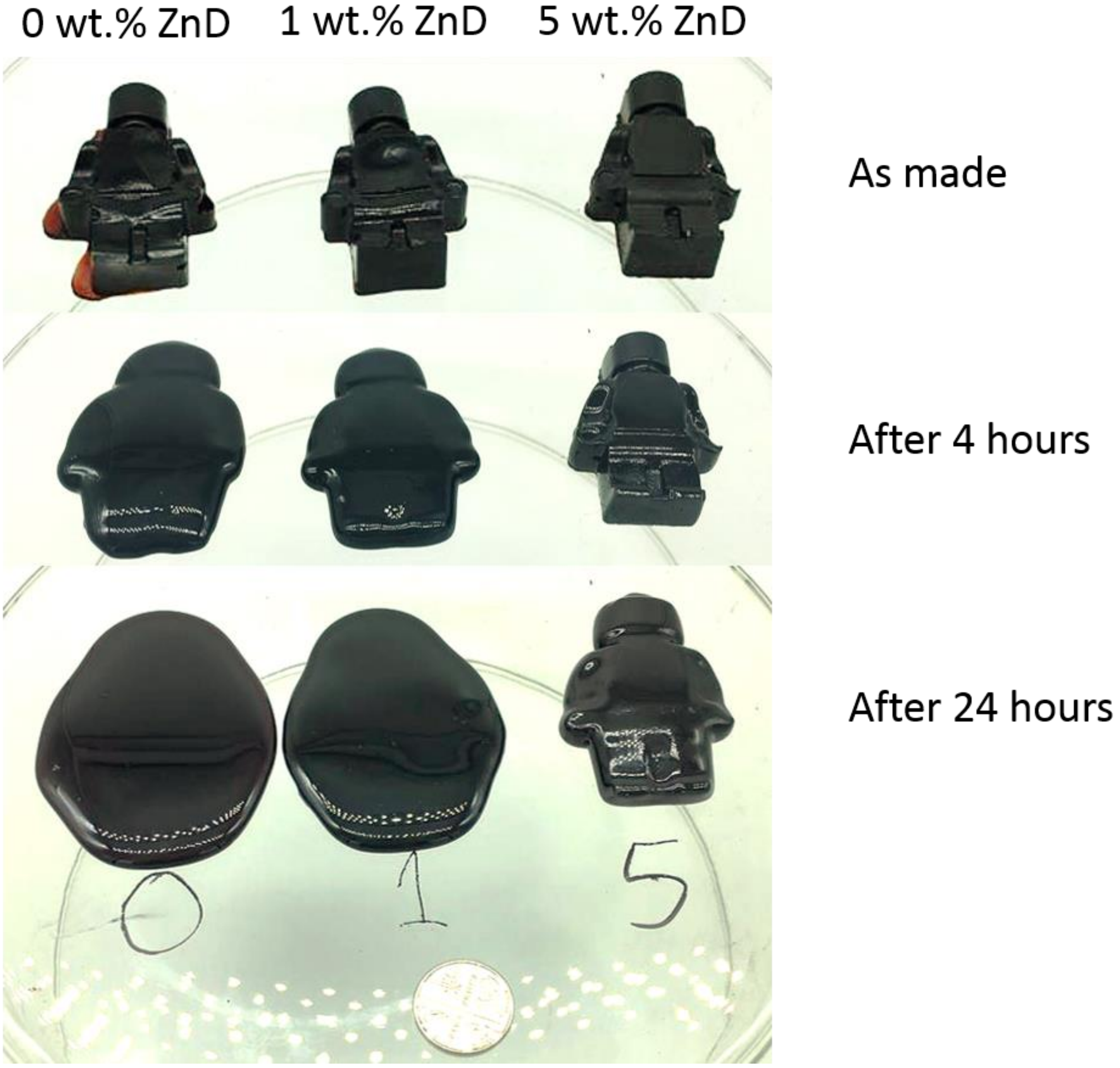

Supplementary figure 34: S-Limonene polymers made by reacting an equal mass of sulfur with limonene, with and without catalyst (Zn Diethyldithiocarbamate). The reaction was performed following the method reported by Chalker *et al*,[3] only adjusting the catalyst loading. Limonene is known to undergo hydrogen abstraction, form by-products including cymene, and produce only low molecular weight polysulfides rather than high molecular weight polymers, and consequently these are not shape persistent.[3, 4] The catalysed samples were notably less tacky to touch, and deformed less with general handling. All samples shower creep under the action of gravity alone, though this was reduced for the catalysed samples.

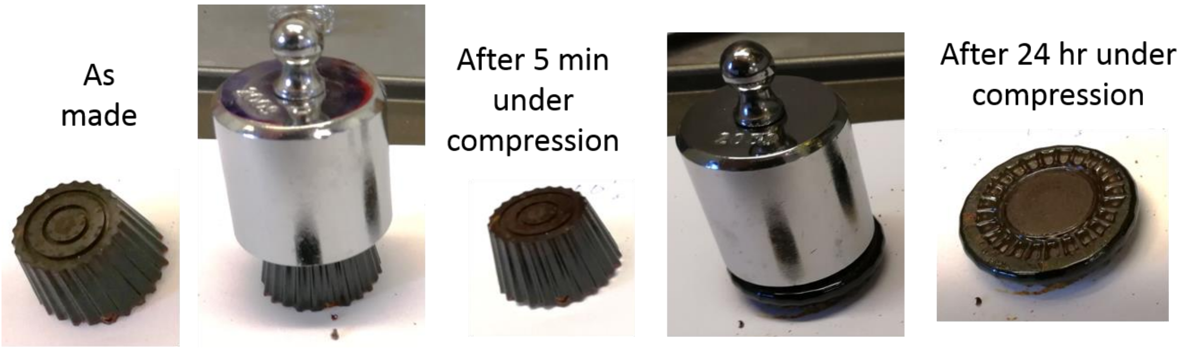

Supplementary figure 35: S-Limonene polymer catalyzed by Zn Diethyldithiocarbamate is able to undergo compression with a 200 g weight, with no noticeable effect after 5 minutes. However, if left under compression for 24 hours the sample is noticeably deformed. Un-catalysed samples are fully deformed within 5 minutes under compression.

GPC of soluble polymers:

The nature of the inverse vulcanization reaction leads to crosslinked polymers, and as such most of the polymers reported here are insoluble. Only two of the polymers, S-limonene and S-TVCH, were found to be fully soluble. These we dissolved in chloroform (10 mg/mL) to allow analysis by GPC.

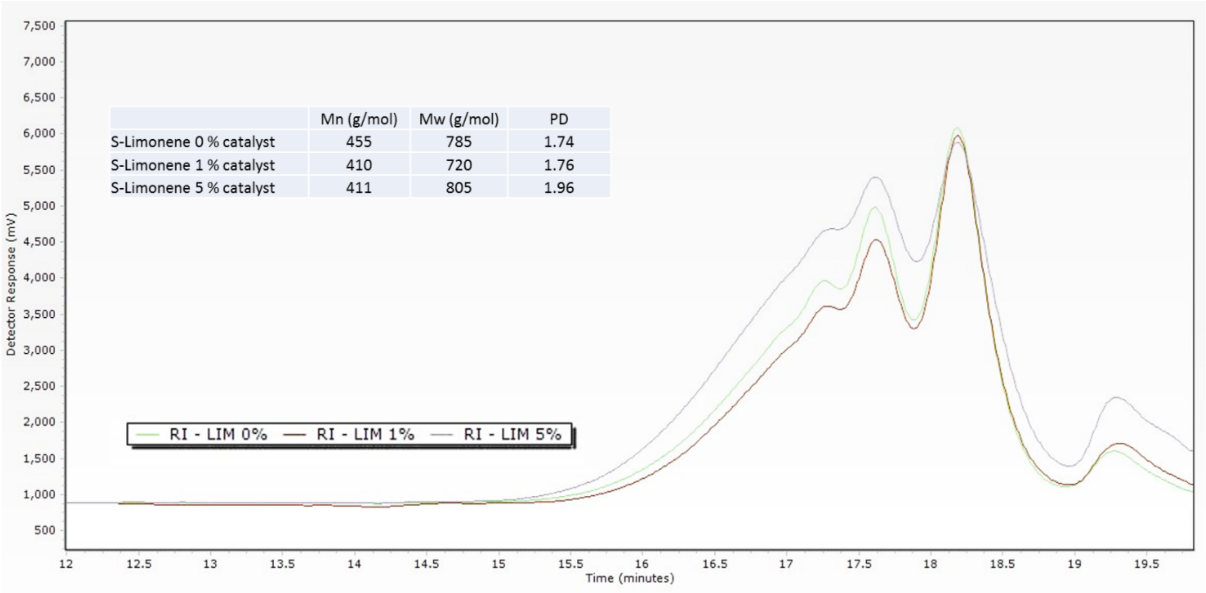

Supplementary figure 36. GPC chromatograms, of the refractive index signal as a function of retention time, for S-limonene polymers with varying ZnD_2 catalyst loadings. There is little difference between the 0 and 1 wt.% catalyst loaded samples. However, the sample loaded with 5 wt.% catalyst has a broader distribution of molecular weights. The inclusion of higher molecular weight material may account for the reduced creep of the 5 wt.% catalyst sample, as

well as its higher T_g. Mn and Mw numbers given in the inset are by comparison to conventional linear polystyrene standard calibration, and are provided for comparative purposes, but should not be taken as accurate as this is likely a poor model for these polymers.

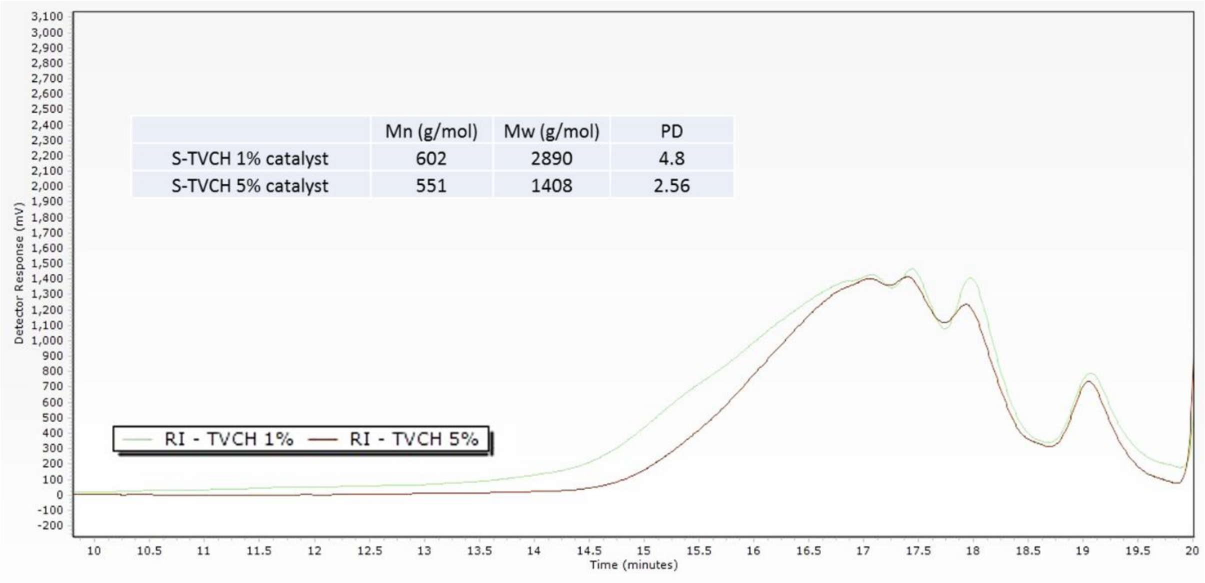

	Mn (g/mol)	Mw (g/mol)	PD
S-TVCH 1% catalyst	602	2890	4.8
S-TVCH 5% catalyst	551	1408	2.56

Supplementary figure 37. GPC chromatograms, of the refractive index signal as a function of retention time, for S-limonene polymers with varying ZnD_2 catalyst loadings. Both samples can be seen to have significantly higher molecular weight than S-limonene. This likely accounts for why S-TVCH displays greater shape persistency (non-creeping solid) in comparison to S-limonene (creeping high viscous liquid), despite both being soluble. The lower catalyst loading sample shows a higher molecular weight, which is consistent with DSC findings that show it also has a higher T_g. Mn and Mw numbers given in the inset are by comparison to conventional linear polystyrene standard calibration, and are provided for comparative purposes, but should not be taken as accurate as this is likely a poor model for these polymers.

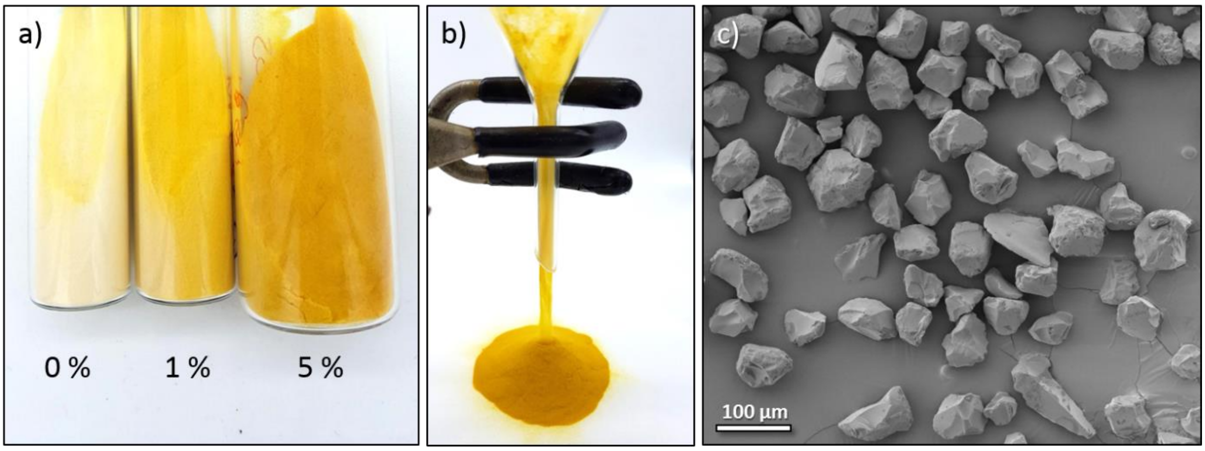

Supplementary figure 38. a) Photograph of powders of silica gel coated with an additional 10 wt.% loading of sulfur-limonene copolymer synthesized using 0 wt.% (left), 1 wt.% (middle), and 5 wt.% ZnD_2 catalyst (right). The colour is noticeably darker with more catalyst. b) Photograph of the polymer coated silica gel flowing through a funnel as a free flowing powder. c) SEM image of the particles after coating with polymer.

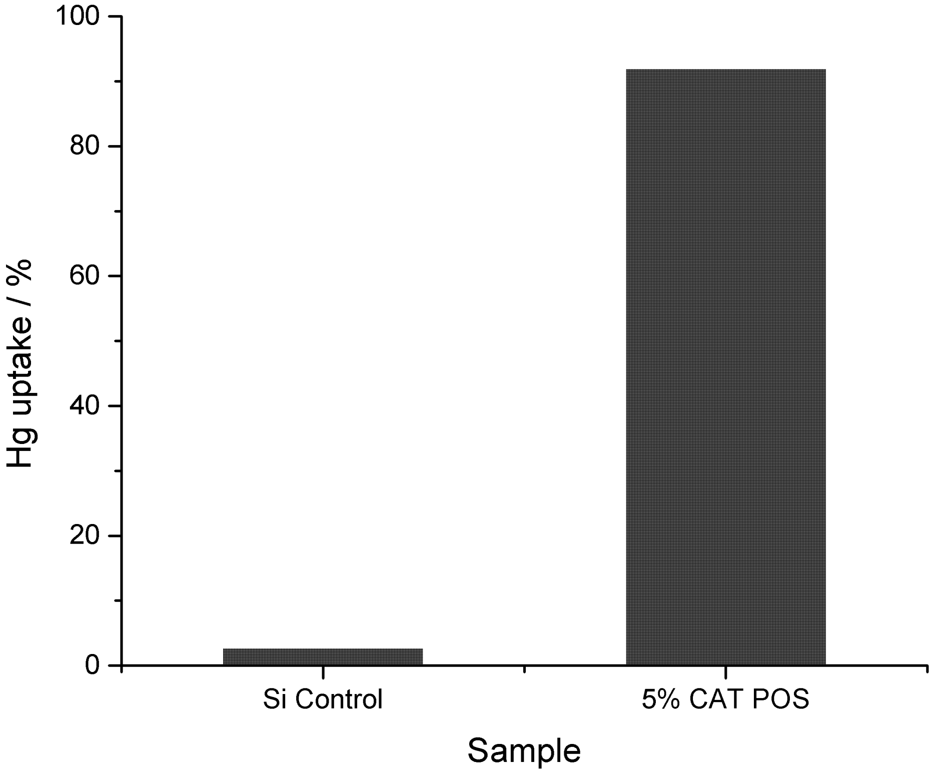

Supplementary figure 39: Uptake of Hg from a 1000 ppm aqueous solution of $HgCl_2$ by uncoated silica compared to silica coated with sulfur-limonene copolymer (10 wt.% loading).

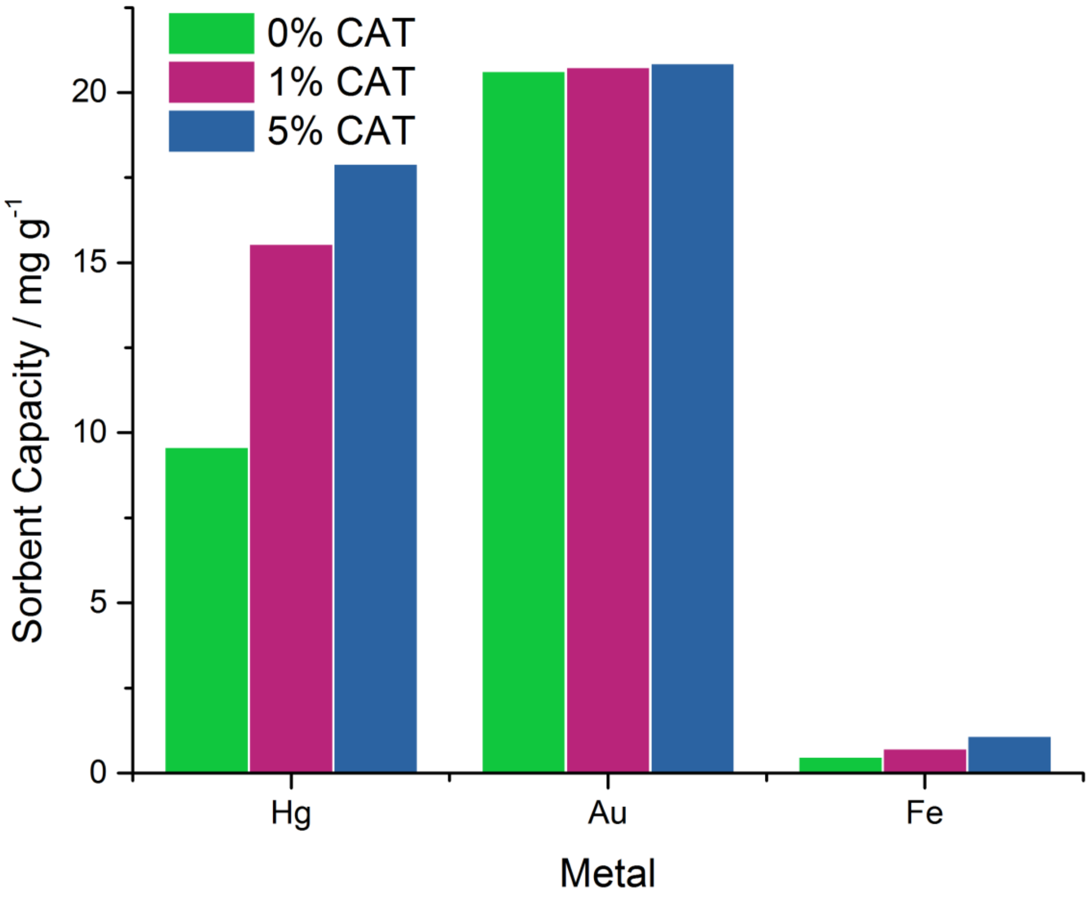

Supplementary figure 40: Uptake of metal by sulfur-limonene coated silica gel from 400 ppm aqueous solutions of mercury chloride, iron chloride, and gold chloride, with varying ZnD_2 catalyst loading, after 1 hour. At this concentration the polymer was able to almost quantitatively remove the gold from solution, hence the necessity to increase the gold concentration to 800 ppm (as shown in Fig. 3 of the main paper), to more accurately represent the capacity. It can be seen that the affinity of the polymer for soft metal ions such as Hg and Au is significantly higher than for the harder ion of Fe.

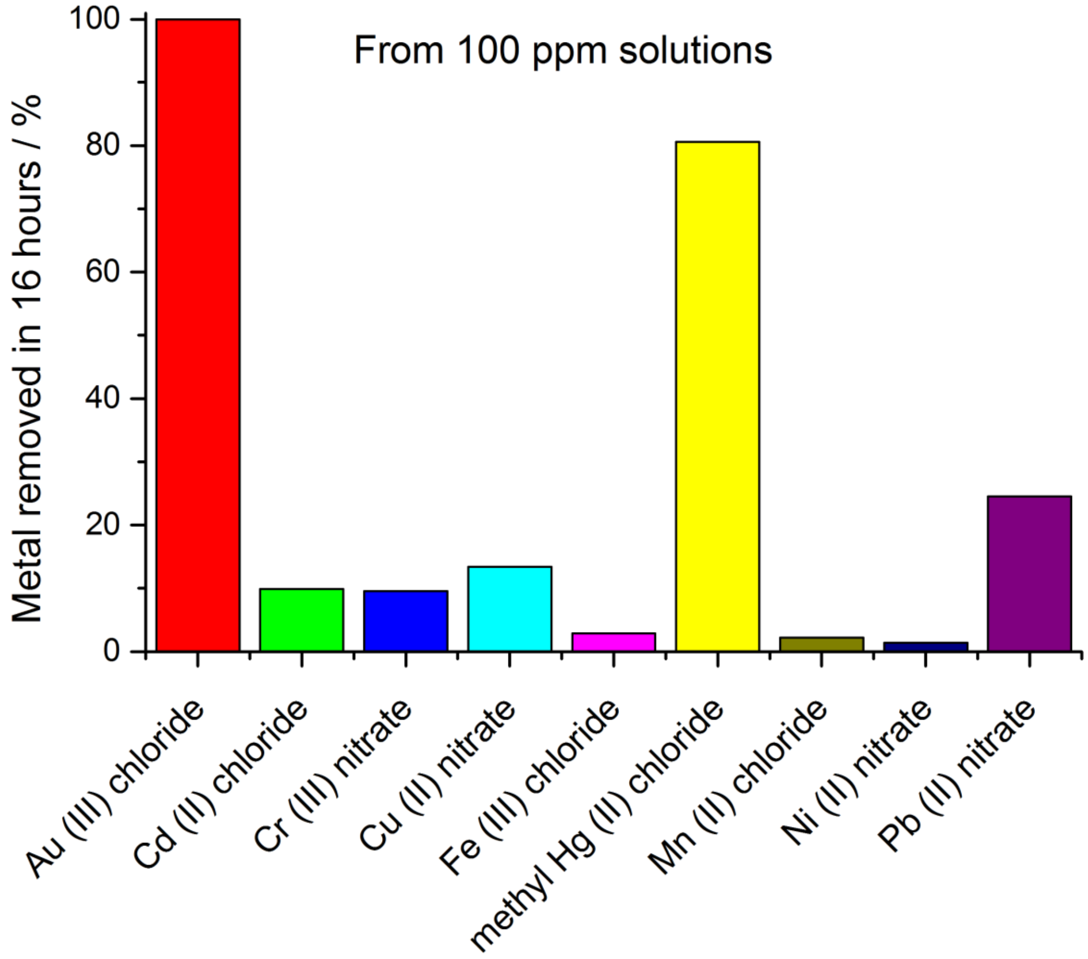

Supplementary figure 41: Uptake of metal by sulfur-limonene coated silica gel from 100 ppm aqueous solutions of various metals, after 16 hours. The affinity for gold and mercury can be seen to be considerably higher than for many other metals. It is significant that the polymer shows good affinity for mercury in the form of methyl mercury chloride, as well as $HgCl_2$. Methyl mercury chloride is the form in which mercury is most commonly found in environmental pollution, and contamination of land and waterways. It is in this form that there is most need for environmental remediation, and methyl mercury chloride is commonly seen as more challenging to adsorb than mercury chloride.[5]

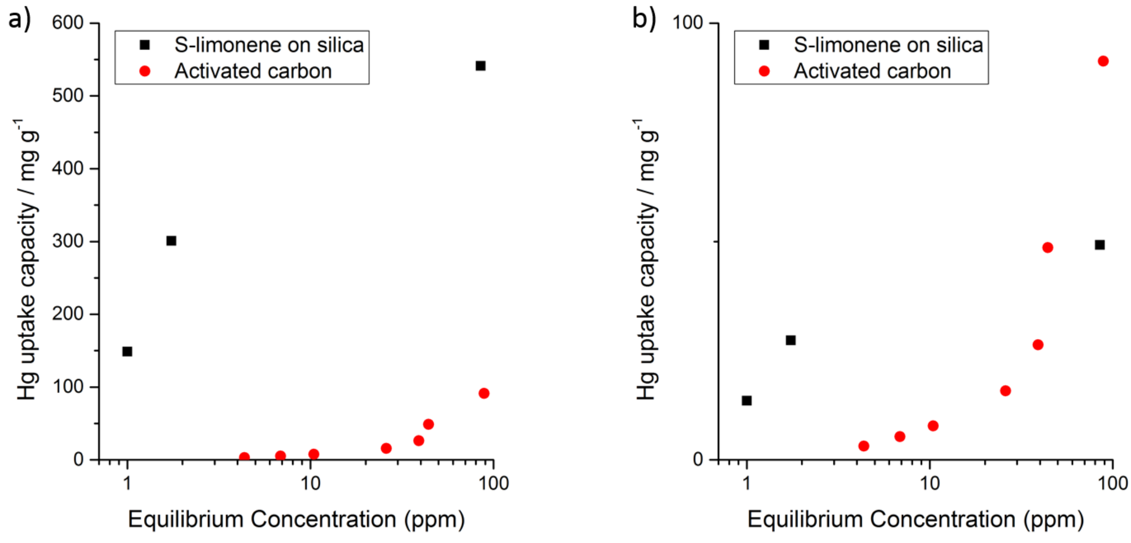

Supplementary figure 42: Mercury uptake isotherms, from aqueous solutions of $HgCl_2$, by S-limonene copolymers compared to commercial activated carbon.[6] a) Hg uptake into S-limonene coated on silica gel calculated from the mass of polymer only. b) Hg uptake into S-limonene coated on silica gel calculated from the total mass of sorbent (polymer and silica). The high sulfur polymer has a much higher affinity than activated carbon for mercury at industrially relevant low concentrations (e.g. 5 ppm and below). Even when considering the total sorbent mass, the polymer-on-silica outperforms commercial activated carbon (commonly used industrially) up to about 40 ppm concentration.

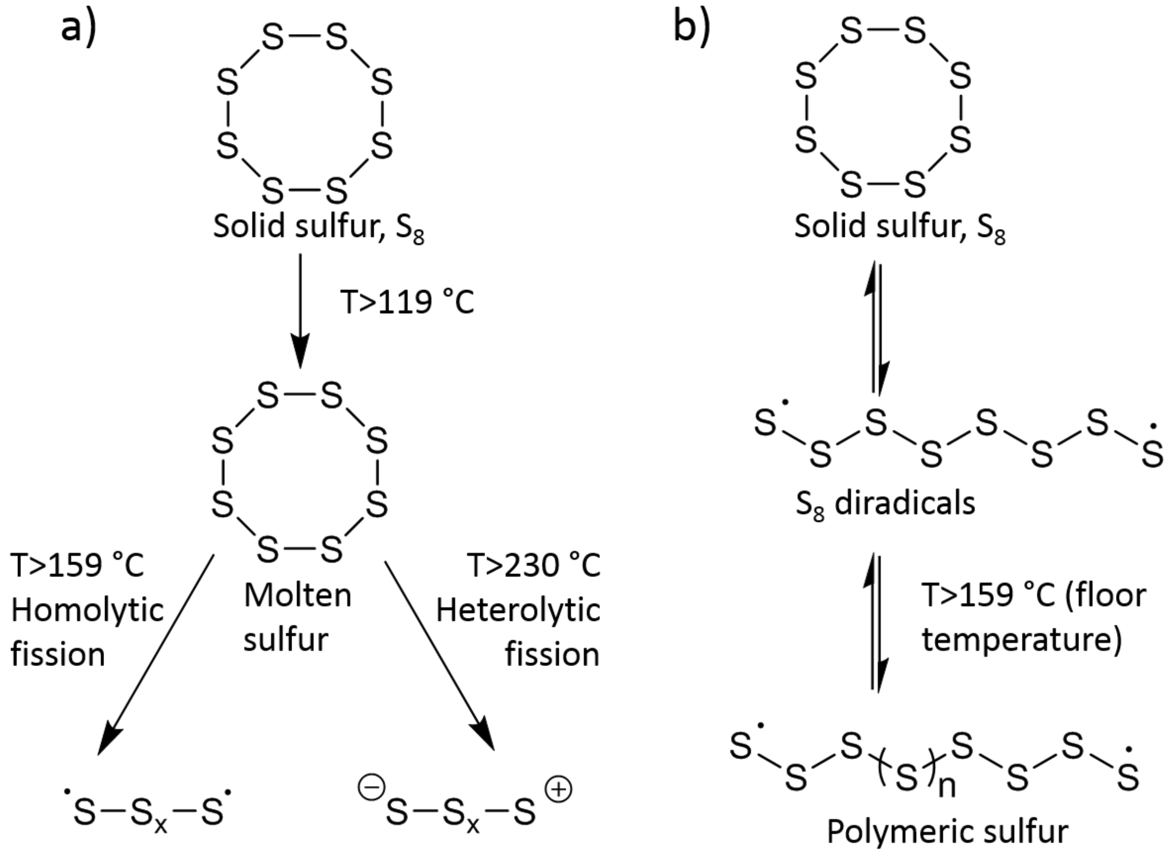

Supplementary figure 43 a) Potential cleavages of sulfur, either homolytically or heterolytically. b) Reversible procedure of ring opening/closing and polymerization/depolymerization of sulfur. The "floor temperature" is the temperature below which no polymeric species are found in appreciable quantities.[7]

Supplementary table 6. Reaction conditions for a series of S-DCPD reactions monitored by NMR in deuterated chloroform. All entries 50 wt.% each of sulfur and crosslinker, except entry number #1, which is pure DCPD prior to reaction. The numbers on the left hand side correspond to the labelling of NMR spectra in the following Supplementary figures. Labels on the second column correspond to the labelling in the photographic images presented in the supplementary figure 44 below. NMRs are presented in supplementary figures 45-52.

No.	Sample name	Catalyst	Heating	Heating time
17	L6	No	Initially 185 °C, dropped to 135 °C after 10 minutes	3 hours
16	L5			1 hour
15	L4			10 min
14	L3			7 min
13	L2			4 min
12	L1			1 min
11	C5	1 wt % ZnD_2	135 °C	3 hours
10	C4			1 hour
9	C3			20 min
8	C2			10 min
7	C1			1 min
6	D5	No	135 °C	40 min
5	D4			30 min
4	D3			20 min
3	D2			10 min
2	D1			1 min
1	D	No	rt	0 min

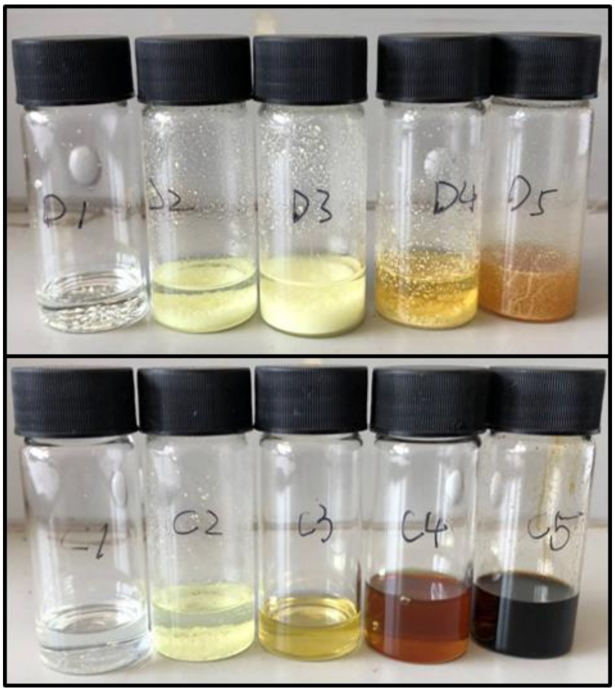

Supplementary figure 44. Photographs of reactions D1-D5 (above) and C1-C5 (below), after cooling to room temperature.

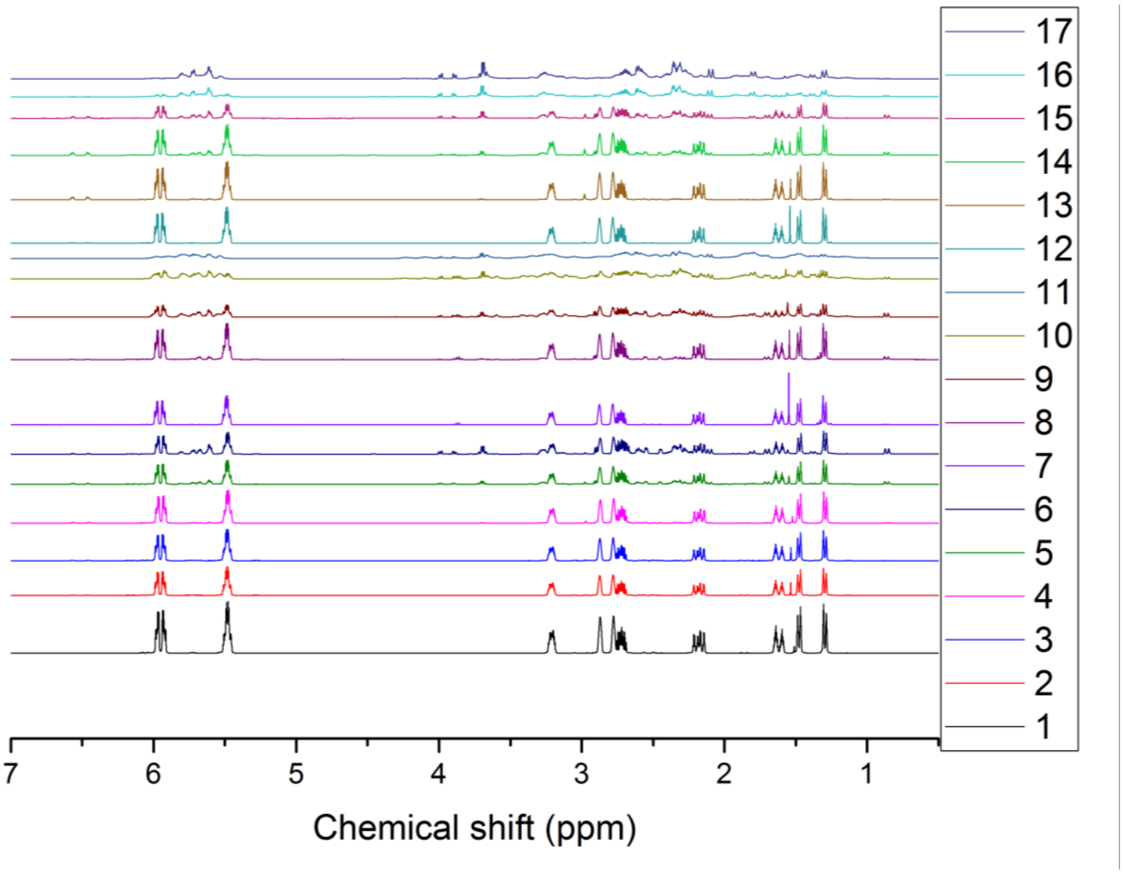

Supplementary figure 45. ^{1}H NMR (in $CDCl_3$), of sulfur-DCPD after reaction times and conditions as listed in Supplementary table 4.

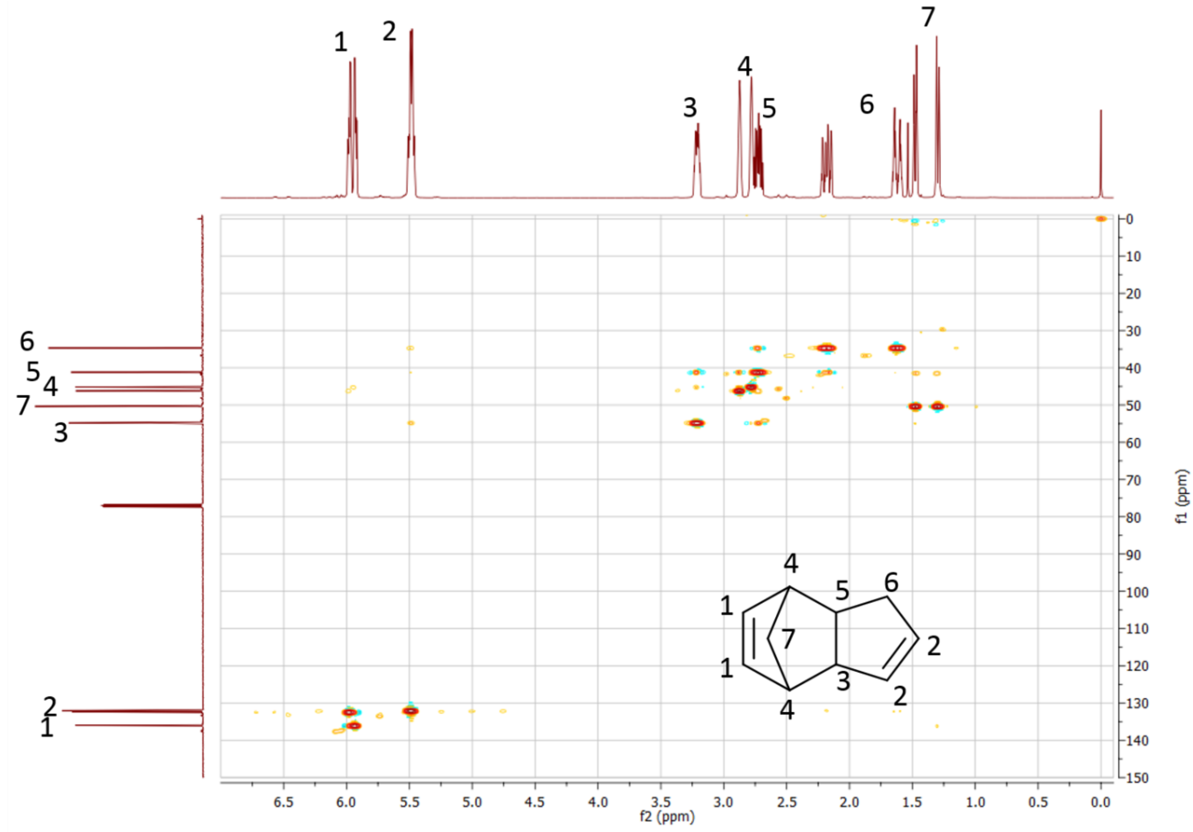

Supplementary figure 46. HSQC NMR of sample #2 from Supplementary table S4. The spectrum shows only unreacted DCPD. The peaks of the ^{1}H (horizontal) and ^{13}C (vertical) spectra are assigned on the structure.

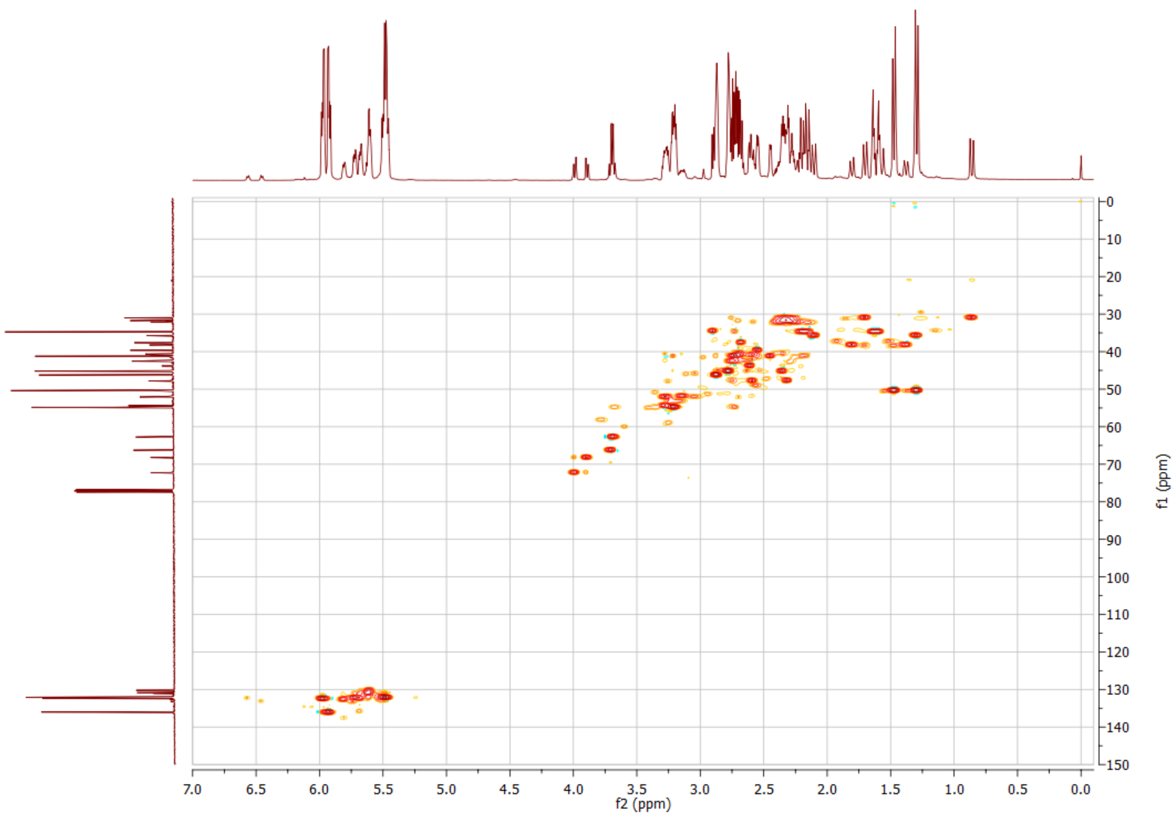

Supplementary figure 47. HSQC NMR of sample #6 from Supplementary table S4, sulfur reacted with DCPD at low temperature (135 °C) for 40 minutes, in the absence of catalyst. Evidence of reaction can be seen in the formation of new peaks in the 3.5 – 4 ppm range associated with S-C-H protons. Changes in the 5.5- 6 ppm region may be associated with shifts to the vinylic protons on one double bond position, after the addition of sulfur across the other C=C bond of the same molecule. However, there is also a signal appearing at ~6.5 ppm, associated with proton substitution at the α position causing a shift to downfield in the vinylic protons.

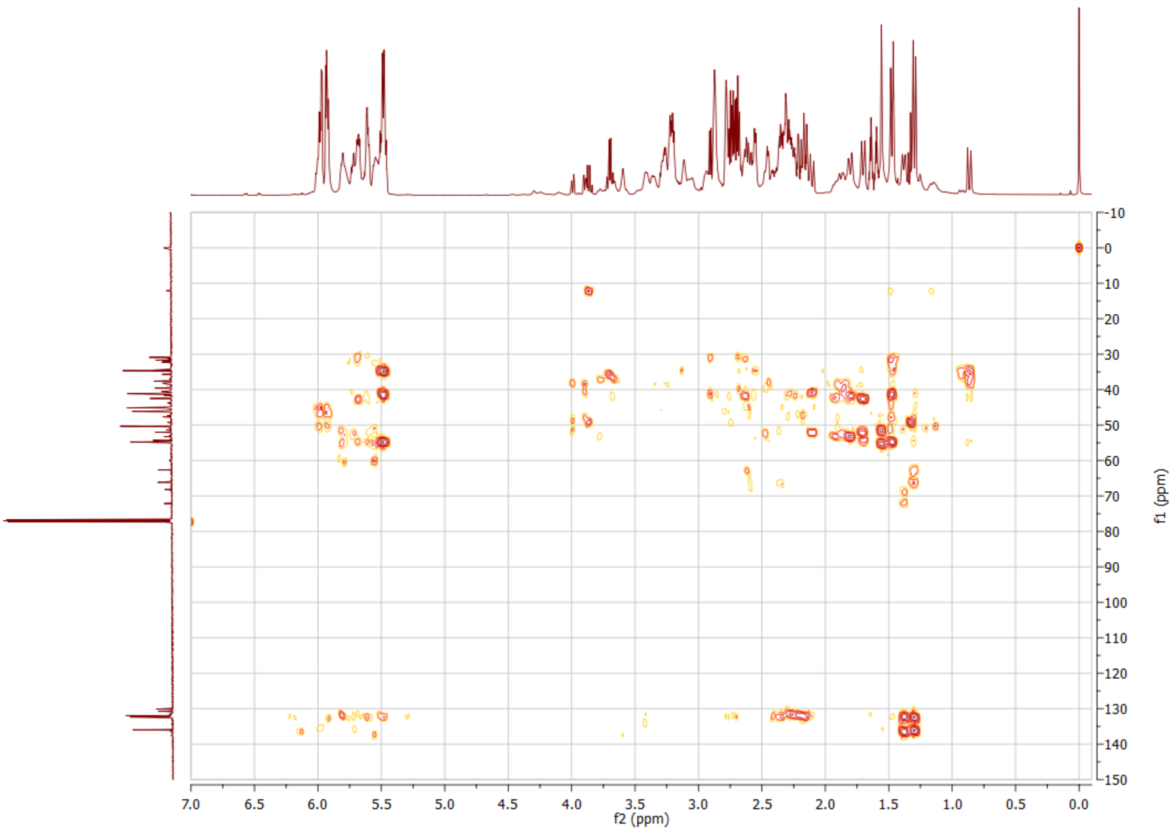

Supplementary figure 48. HSQC NMR of sample #9 from Supplementary table S4, sulfur reacted with DCPD at low temperature (135 °C) for 20 minutes, in the presence of 1 wt.% ZnD_2 catalyst. Evidence of reaction can be seen in the formation of new peaks in the 3.5 – 4 ppm range associated with S-C-H protons, as well as a marked broadening of all peaks, indicative of polymerization. Changes in the 5.5- 6 ppm region may be associated with shifts to the vinylic protons on one double bond position, after the addition of sulfur across the other C=C bond of the same molecule. There is almost no detectible signal appearing at ~6.5 ppm, associated with proton substitution at the α position causing a shift to downfield in the vinylic protons. ZnD_2

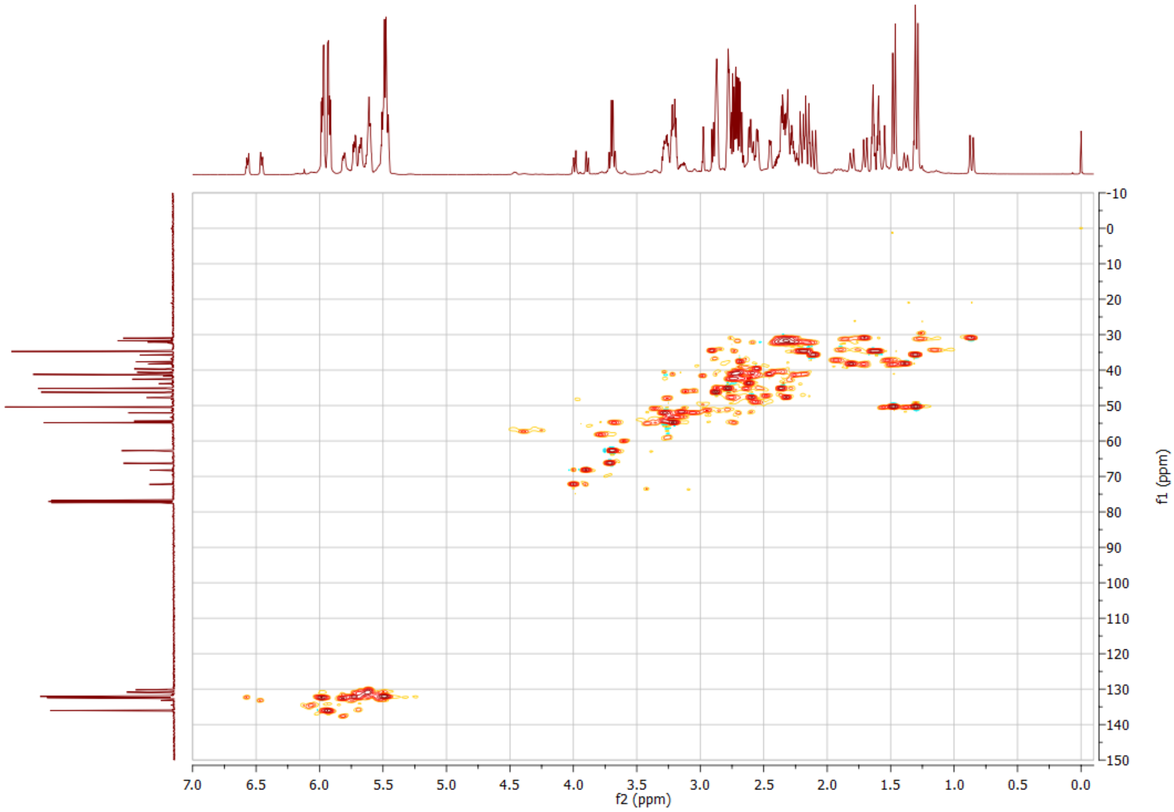

Supplementary figure 49. HSQC NMR of sample #15 from Supplementary table S4, sulfur reacted with DCPD at high temperature (185 °C) for 10 minutes, in the absence of catalyst. Evidence of reaction can be seen in the formation of new peaks in the 3.5 – 4 ppm range associated with S-C-H protons. Changes in the 5.5- 6 ppm region may be associated with shifts to the vinylic protons on one double bond position, after the addition of sulfur across the other C=C bond of the same molecule. However, there is also a significant signal appearing at ~6.5 ppm, associated with proton substitution at the α position causing a shift to downfield in the vinylic protons. This signal is higher than that detected for lower temperature reactions, even at the same degree of reaction.

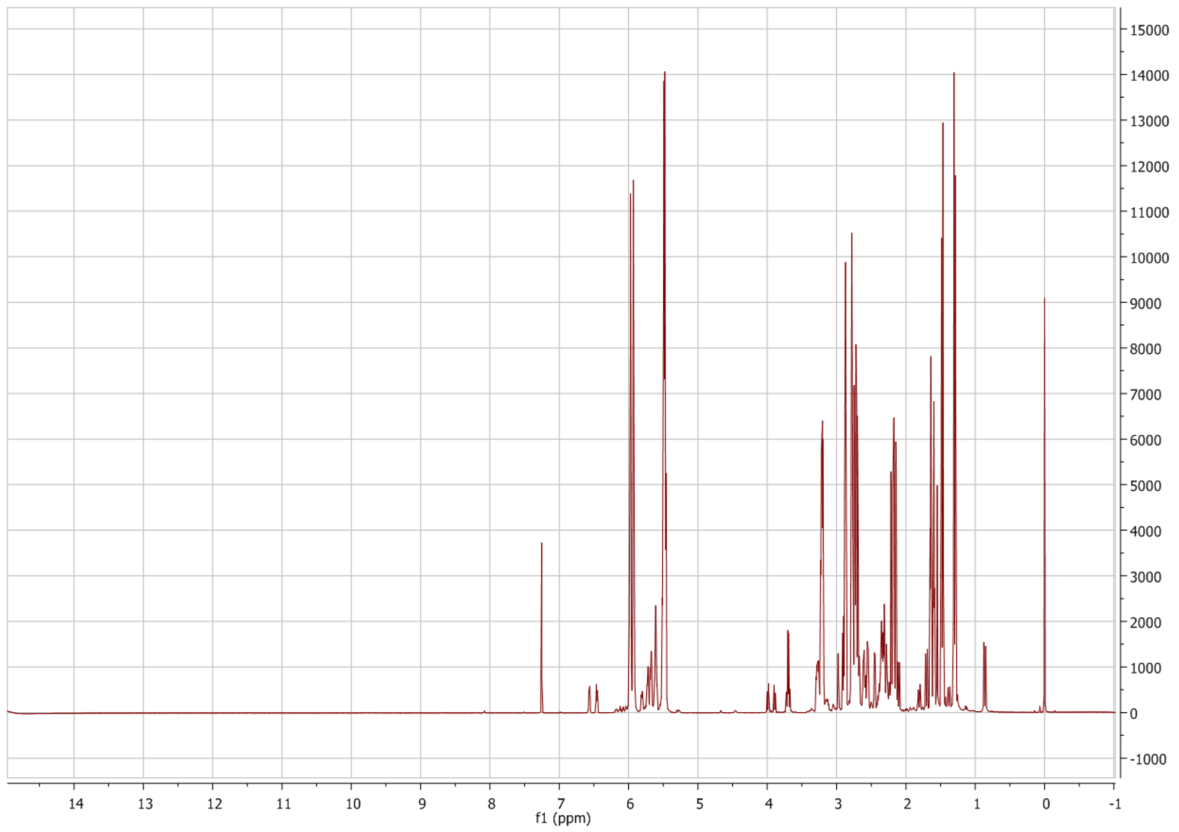

Supplementary figure 50. ^{1}H NMR (in $CDCl_3$), of sulfur-DCPD at low temperature (135 °C), without catalyst, at the reaction time that showed the highest signal at 6.5 ppm (30 minutes).

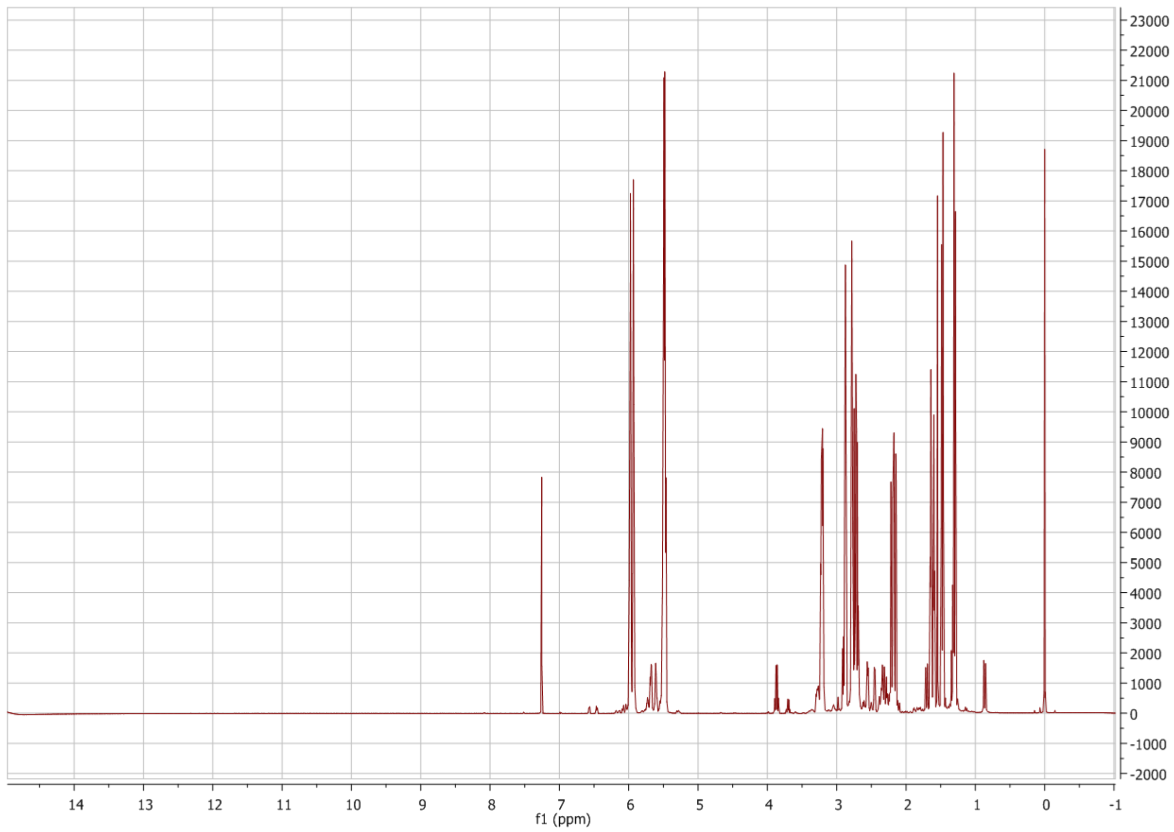

Supplementary figure 51. ^{1}H NMR (in $CDCl_3$), of sulfur-DCPD at low temperature (135 °C), with 1 wt.% ZnD_2 catalyst, at the reaction time that showed the highest signal at 6.5 ppm (10 minutes).

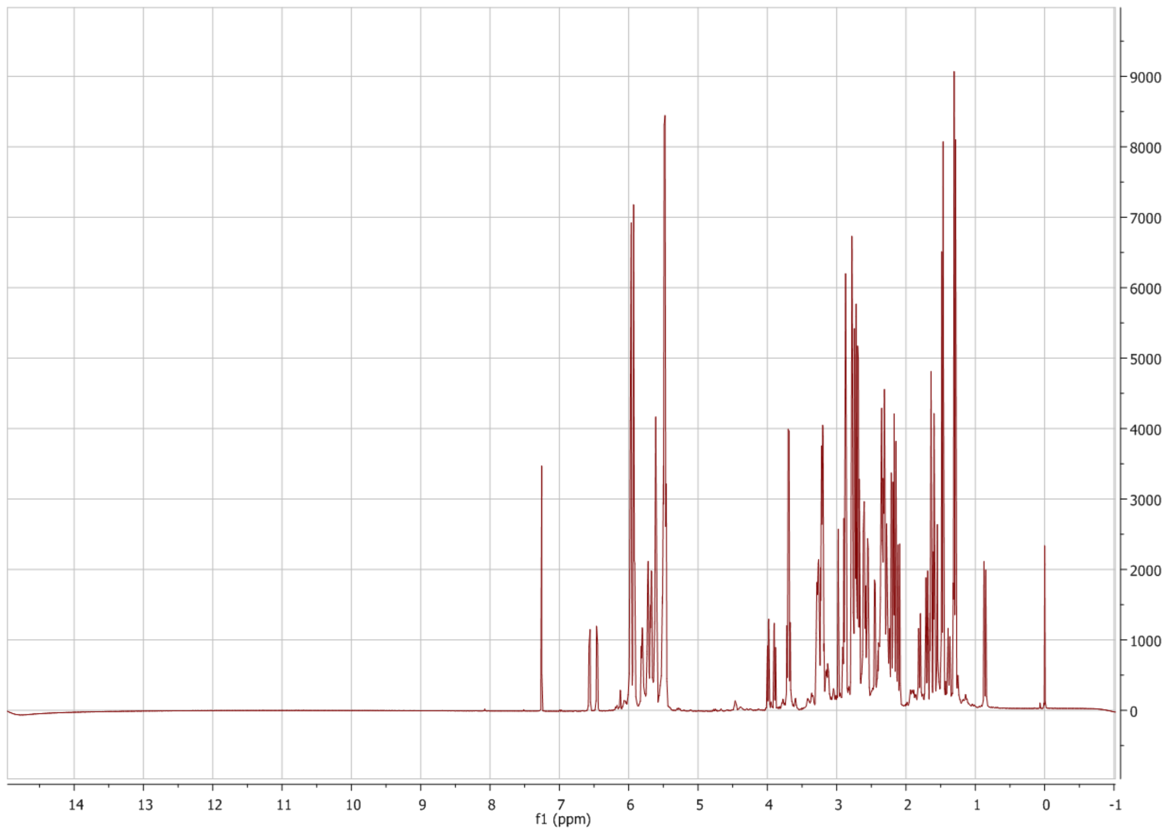

Supplementary figure 52. ^{1}H NMR (in $CDCl_3$), of sulfur-DCPD with high temperature initiation (185 °C), without catalyst, at the reaction time that showed the highest signal at 6.5 ppm (10 minutes).

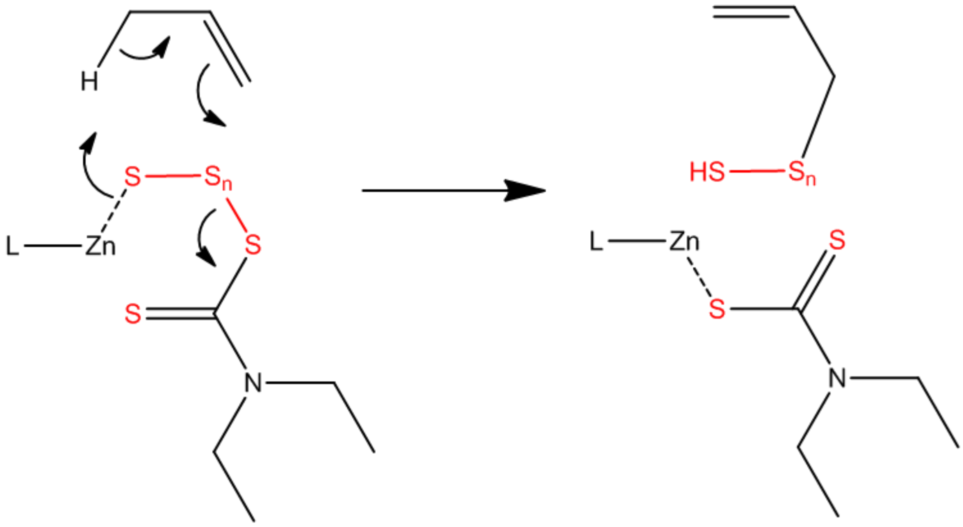

Supplementary figure 53: Proposed mechanism for proton abstraction from the α-position, by sulfur, catalyzed by a ZnD_2 catalyst.[8]

Supplementary References:

1. Hasegawa Y, Maeda M, Nakanishi T, Doi Y, Hinatsu Y, Fujita K*, et al.* Effective Optical Faraday Rotations of Semiconductor EuS Nanocrystals with Paramagnetic Transition-Metal Ions. *Journal of the American Chemical Society* 2013, **135**(7)**:** 2659-2666.

2. Crockett MP, Evans AM, Worthington MJH, Albuquerque IS, Slattery AD, Gibson CT*, et al.* Sulfur-Limonene Polysulfide: A Material Synthesized Entirely from Industrial By-Products and Its Use in Removing Toxic Metals from Water and Soil. *Angewandte Chemie International Edition* 2015, **55**(5)**:** 1714-1718.

3. Crockett MP, Evans AM, Worthington MJH, Albuquerque IS, Slattery AD, Gibson CT*, et al.* Sulfur-Limonene Polysulfide: A Material Synthesized Entirely from Industrial By-Products and Its Use in Removing Toxic Metals from Water and Soil. *Angewandte Chemie International Edition* 2016, **55**(5)**:** 1714-1718.

4. Parker DJ, Jones HA, Petcher S, Cervini L, Griffin JM, Akhtar R*, et al.* Low cost and renewable sulfur-polymers by inverse vulcanisation, and their potential for mercury capture. *Journal of Materials Chemistry A* 2017, **5**(23)**:** 11682-11692.

5. Esdaile LJ, Chalker JM. The Mercury Problem in Artisanal and Small-Scale Gold Mining. *Chemistry – A European Journal* 2018, **24**(27)**:** 6905-6916.

6. Lee JSM, Parker DJ, Cooper AI, Hasell T. High surface area sulfur-doped microporous carbons from inverse vulcanised polymers. *Journal of Materials Chemistry A* 2017, **5**(35)**:** 18603-18609.

7. Tobolsky AV, MacKnight W, Beevers RB, Gupta VD. The glass transition temperature of polymeric sulphur. *Polymer* 1963, **4:** 423-427.

8. Nieuwenhuizen PJ, Ehlers AW, Haasnoot JG, Janse SR, Reedijk J, Baerends EJ. The Mechanism of Zinc(II)-Dithiocarbamate-Accelerated Vulcanization Uncovered; Theoretical and Experimental Evidence. *Journal of the American Chemical Society* 1999, **121**(1)**:** 163-168.
